# Proton and alpha radiation-induced mutational profiles in human cells

**DOI:** 10.1038/s41598-023-36845-3

**Published:** 2023-06-16

**Authors:** Tiffany M. Delhomme, Maia Munteanu, Manuela Buonanno, Veljko Grilj, Josep Biayna, Fran Supek

**Affiliations:** 1 Genome Data Science, Institute for Research in Biomedicine (IRB Barcelona), The Barcelona Institute of Science and Technology (BIST), Barcelona, Spain; 2 Radiological Research Accelerator Facility (RARAF), Columbia University, New York, USA; 3 Catalan Institution for Research and Advanced Studies (ICREA), Barcelona, Spain

**Keywords:** Genomic instability, Mutation, Genome informatics, Genomics

## Abstract

Ionizing radiation is known to be DNA damaging and mutagenic, however less is known about which mutational footprints result from exposures of human cells to different types of radiation. We were interested in the mutagenic effects of particle radiation exposures on genomes of various human cell types, in order to gauge the genotoxic risks of galactic cosmic radiation, and of certain types of tumor radiotherapy. To this end, we exposed cultured cell lines from the human blood, breast and lung to fractionated proton and alpha particle (helium nuclei) beams at doses sufficient to considerably affect cell viability. Whole-genome sequencing revealed that mutation rates were not overall markedly increased upon proton and alpha exposures. However, there were modest changes in mutation spectra and distributions, such as the increases in clustered mutations and of certain types of indels and structural variants. The spectrum of mutagenic effects of particle beams may be cell-type and/or genetic background specific. Overall, the mutational effects of repeated exposures to proton and alpha radiation on human cells in culture appear subtle, however further work is warranted to understand effects of long-term exposures on various human tissues.

## Introduction

Humans have a keen interest in travelling in space, from deep space exploration to extraterrestrial colonization. In addition, the space environment was also considered for biomedical research purposes, for example the “tumors in space” experiment aims to study the effects of microgravity and exposure to space radiation on tumor organoids^1^. Spaceflight is usually defined as crossing the Kármán separation line i.e. 80–100 km altitude above the sea level^2–4^. Above this boundary, the environment dramatically changes, notably in terms of microgravity, temperature, and radiation^5^. Various pathological consequences on different human organ systems arise from travelling in space, including cardiovascular, musculoskeletal, and immune changes^6^. However, a better understanding of space travel impact at the cellular and sub-cellular levels is needed, for instance how the genome integrity is affected.

Important effects of space travel are exerted by the various types of radiation to which tissues are exposed outside the Earth’s atmosphere and magnetosphere. Those include the solar radiation, the radioactive environment of the planet, and galactic cosmic radiation (or rays; GCR), which originates mainly from the Sun or from outside of the Solar system but within the Milky Way galaxy (there exist also the extra-galactic cosmic radiations, which are less relevant as a hazard due to low flux and extremely high energies). The GCR encompasses photon-based radiation as well as particle radiations, including neutrons and charged particles; among the latter, protons and helium nuclei are the most abundant. Cosmic radiation has emerged as an issue of concern, since exposure to radiation may increase cancer risk in astronauts^7,8^. A study of the mutagenic mechanisms of different cosmic radiation types upon the human genome would be helpful to understand the basis of the increased risk of cancer or of reproductive harm resulting from space travel.

Photons are the better-studied component of the GCR, since they consist of gamma rays and X-rays, whose biological impact was studied in other contexts. For instance, gamma rays were released during the Chernobyl nuclear power plant disaster. The genomic profiles of thyroid carcinomas in irradiated children post-Chernobyl contained similar driver mutations and gene fusions as in non-radiation-associated thyroid tumors^9^. However, there was a radiation dose-dependent increase of fusion driver events, as well as more generally of certain types of structural variants (rearrangements)^10^. Consistent with this mutational impact that can generate somatic driver mutations, ionizing radiation (IR)-generating incidents (e.g. the Chernobyl disaster, and also the Hiroshima and Nagasaki A-bomb attacks) also increased cancer risk proportionally to the dose of exposure^11,12^.

In addition to nuclear incidents, radiation exposures can result from medical uses. Commonly they involve X-rays, used for both diagnostic purposes, and tumor radiotherapy. The mutational footprint of therapeutic X-ray exposure was examined in the genomes of radiation-associated second malignancies and of post-treatment metastatic tumors, reporting an increase of small and large deletions, and also certain mutational signatures^13,14^. For instance, this entails a particular signature of indels (the PCAWG Signature ID8) characterized by larger deletions (≥ 5-bp) without flanking micro-homology, previously linked to double-strand break repair^15^. Consistently with their mutagenic effect, medical use of X-rays has been reported to increase cancer risk^16^.

Regardless of the source of the radiation—either the cosmos, nuclear accidents or medical uses—it is not clear if IR consisting of charged particles, such as protons or alpha beams, would have similar mutagenic effects to more commonly studied photons (X-rays and gamma-rays). Generally, all types of IR, including gamma-rays, X-rays, energetic charged particles [protons, alpha particles (helium nuclei) and heavier ions} and neutrons have been classified as human carcinogens by the World Health Organization (WHO)^17^. This suggests mutagenic impacts of various radiation types, however research on the specific mutational footprints of charged particles on human genome stability has been limited.

Mutagenesis induced by particulate radiation is of interest because during transit beyond low Earth orbit, every cell nucleus within an astronaut’s body is traversed, on average, by an energetic proton every few days, and by a helium nucleus once every few weeks. The mutational effect of proton radiation is of interest also for medical reasons, as protons are increasingly adopted in the clinic to treat cancer^18^, and appear promising with respect to toxicity profiles^19–22^. Proton therapy may reduce the health risks (compared to X-rays) of secondary malignancies^23^, and of severe lymphopenia^24,25^. The above provides motivation for studying the mutagenic effects of protons, and of helium nuclei (alpha) radiation, which have the potential to result in long term health effects in exposed individuals.

The activity of mutational processes in DNA—which may arise from both endogenous factors such as DNA repair deficiencies and exogenous factors such as tobacco smoking—can be captured by mutational signatures^26^, mathematical constructs that describe the differential frequencies of mutation types across many genomes. Such signatures are commonly based on trinucleotide spectra of single nucleotide variants (SNVs), however they can also be based on small insertions and deletions (indels), structural variants (rearrangements), or mutation clusters^15,27,28^. SNV and indel mutational signatures in human cancer are well characterized and organized in comprehensive catalogues^15,29^, including signatures in tumors pre-treated with photon IR^13,14,30^.

In addition, some genomic studies have reported mutational signatures on experimental models, including experimentally-induced mutational signatures of (non-ionizing) UV radiation in human cell lines^31^, and also in cultured cells from healthy human tissues^32,33^. Additionally, signatures of IR were studied in exposed mice^34,35^ and in the worm *C. elegans*^36^; both reported SNV signatures enriched in C>T transitions^15^.

To increase our understanding of the potential impact of particle radiation encountered during space travel on human genome integrity, it is important to systematically examine the DNA mutations arising from the exposure in cells originating from different human tissues. To this end, we analyzed the whole genome sequences of three human cancer cell lines, A549 (lung adenocarcinoma, epithelial cells), HAP1 (chronic myelogenous leukaemia, blood cells) and MCF7 (breast carcinoma, epithelial cells). Those cell lines were irradiated repeatedly with two types of particle radiations—proton or helium fluxes—in order to identify the mutational footprints induced and their potential cell type-specificity.

## Results

### Determining proton and helium ion fraction, and irradiating the cell lines

**Figure 1:**
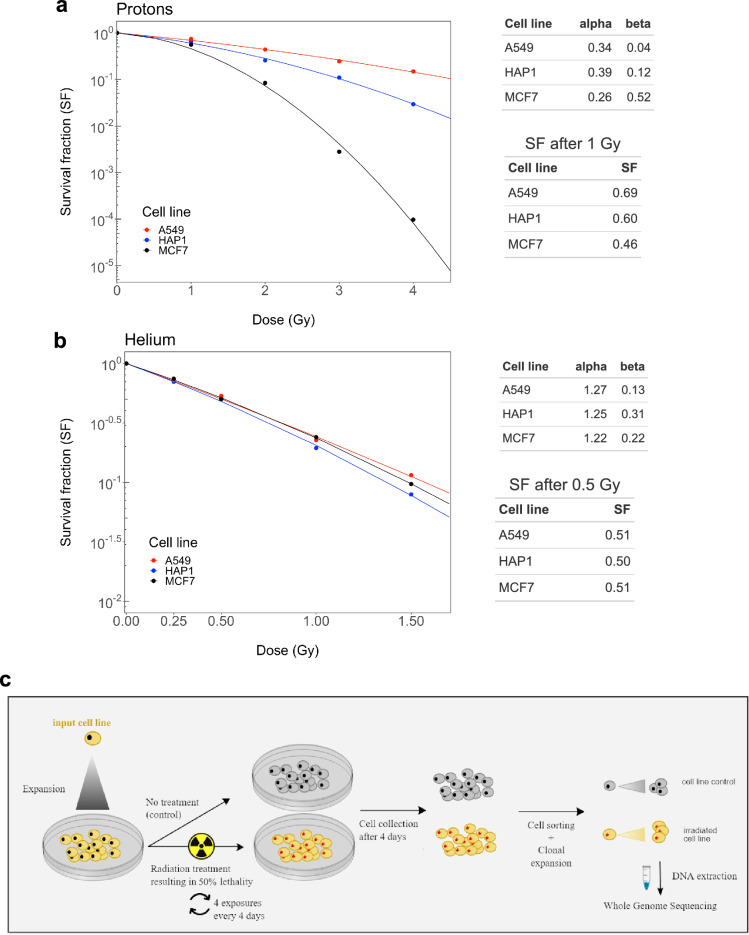
Overview of the experimental design. (**a**,**b**) Experimental identification of radiation dose in order to achieve ~ 50% of cell lethality (i.e., ~ 50% of cell survival) independently across the three cell lines and across the two types of radiations. SF, survival fraction. (**c**) Schematic overview of the experimental design of the study. A549, MCF7 and HAP-1 cell lines were exposed to GCR (protons or helium ions). *GCR* Galactic Cosmic Radiation. Single cells from treated and untreated conditions were separated by fluorescence-activated cell sorting (FACS) and clonally expanded. DNA from single-cell derived populations was extracted and subjected to whole genome sequencing.

The dosage of radiation was determined experimentally in order to achieve between 40 and 50% lethality (corresponding to 50% to 60% clonogenic survival), independently across the two types of particle beams (Fig. 1a,b).

This led to a dose of 0.5 Gy for helium ions and a dose of 1 Gy for protons. After an expansion of the three cell lines collected (A549, HAP1 and MCF7), the cells were irradiated using the 5.5 MV Singletron accelerator at the Radiological Research Accelerator Facility (RARAF; see “Methods” for details about the procedure, with the corresponding dosimetry). We exposed each cell line four times, once every 4 days to allow cell recovery between each exposure to facilitate mutation accumulation.

After the final latency period of 4 days, the cells were collected, sorted in a 96-well plate, and a clonal expansion was performed to obtain colonies with a near-identical genome to facilitate identifying mutations (Fig. 1c). Finally, the DNA was extracted from those colonies and whole-genome sequenced (WGS) on an Illumina NovaSeq 6000 machine. Details of the following bioinformatics analysis are described in the “Methods” section.

### Overall burden of various mutation types generated by particle radiation

**Figure 2:**
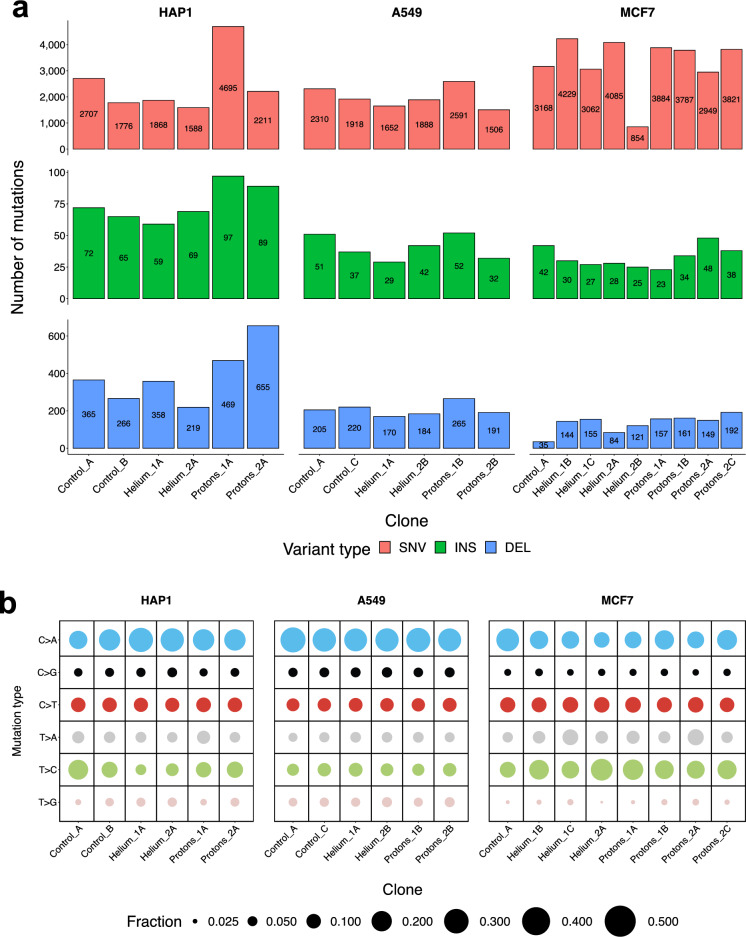
Point mutation counts and classification. (**a**) Distribution of the number of SNVs, small insertions and small deletions per clone in each of the three analyzed cell lines. (**b**) Break-down of point mutations into the 6 main types of mutations (i.e., C>A, C>G, C>T, T>A, T>C, T>G), relative contributions per clone in each cell line.

After the GCR treatments, the cell line genomes contained newly acquired point mutations (SNVs), indels, and structural variants (SVs) in all three cell lines assayed, HAP1, A549 and MCF7, and regardless of the radiation type, protons or helium ions. As a control, we considered clones generated from sham-irradiated (i.e., untreated) cells for all three cell lines; these clones also accumulated certain numbers of SNVs, small indels and SVs, consistent with them undergoing cell divisions and also potentially being exposed to various stresses during handling. The global number of observed mutation events (SNVs and indels) does not markedly differ between conditions (Fig. 2a), suggesting that proton and alpha radiation exposure is not grossly mutagenic to cultured human cells using the fractionated dosing regimen applied herein.

Variant allele fraction (VAF), or the proportion of sequencing reads that contain a particular variant, is a proxy for the proportion of cells in the sequenced population that harbor the mutation. In our experiments, we aimed to select a single treated cell and expand it into a clone, thus the VAFs observed should be centered at 0.5 for heterozygous diploid genome segments. In practice, three different types of VAF patterns were observed (Supp. Fig. 1). One peak at 0.25, which suggests that either two diploid cells were collected at the time of bottlenecking or alternatively, that the genome or its segment may be tetraploid; we observed this commonly for the MCF7 cell line, which is indeed known to be hypertriploid to hypotetraploid^37^. The second pattern is one peak at 0.5 which suggests that one cell was collected and that mutations were found within a diploid segment; this was common for the A549 cells. Finally, a third pattern, a peak at 1, was observed for the HAP1 cell line, as expected due to the unusual genome-wide haploid state of that cell line (Supp. Fig. 1). This cell line can grow as haploid or diploid and may switch spontaneously^38^ and indeed we observed considerable number of VAFs > = 0.5 in HAP1 genomes (Supp. Fig. 1). The VAF distributions in some HAP1 clones e.g., PR1A suggest that a whole-genome doubling may have occurred. We note that one helium-treated MCF7 clone exhibited an unusual VAF distribution, as well as outlying (low) SNV burdens (Fig. 2a), suggesting technical artifacts in this experimental replicate and thus, we excluded the WGS data from this MCF7 sample from further display (whilst still retaining it in the NMF analyses, see below).

### Mutation burdens resulting from proton exposures or putative DNA repair failures

We detected higher amounts of SNVs in the MCF7 cell line (roughly double) than in the two other cell lines after exposure to either particle as well as in the MCF7 untreated cells (Fig. 2a). This may indicate a mutator phenotype in our MCF7 cell line, possibly due to a DNA mismatch repair (MMR) failure (see mutation signature analysis below). However, this is difficult to ascertain since the absolute number of mutations between cell lines/conditions cannot be compared precisely, due to a potentially variable number of cell cycles the cells have undergone during the experiment. On a related note, we further observed an increased number of indels, particularly deletions, in the HAP1 cell line compared to other two cell lines (Fig. 2a). Because this was also observed in the control cells, it indicates that our HAP1 cells have an intrinsically higher rate of indel accumulation, often 1 nt deletions at homopolymers, suggesting a type of microsatellite instability.

To comprehensively assess the differences in mutation burden between different conditions, we implemented a randomization test (see “Methods” for details, Supp. Fig. 2). In all irradiated cell lines, specifically the proton treatment generated the clone with the highest number of indels in its genome, compared to the other conditions (Fig. 2a). This trend is also visible in the randomization results, where the proton-treated clones have more indels compared to helium and untreated groups (unadjusted p = 0.078, p = 0.273), especially clear in the HAP1 cell line (unadjusted p = 0.047, p = 0.031; not statistically significant after FDR correction). For two out of three cell lines, the proton treatment also generated the clone with the highest number of SNVs and generally appeared to generate more SNVs across all cell lines compared to the other treatment groups (p = 0.096 for control, p = 0.074 for the helium comparison). The indel-enrichment trend for proton-treated clones was more prominent for deletions than for insertions (Fig. 2a, Supp. Fig. 2, panel “IDs_del_ins_ratio” p = 0.055). An enrichment of deletions, as we observed in the proton-treated clones, has recently been highlighted in IR-associated tumors^13,14^ which were treated with photon radiation, typically X-rays. The globally most-mutated clone is a single proton-irradiated sample of the HAP1 cell line, which contains 4695 SNVs (Fig. 2a), compared to 2707 and 1776 in the control (non-irradiated) HAP1 samples. Proton exposures also generated the highest number of insertions in the HAP1 cell line (97 and 89 detected in the two proton-treated replicates, compared to 72 in the control), and deletions (469 and 655 detected in proton-treated, compared to 365 in the controls). For this type of alteration, there was a more modest impact of the proton treatment in the A549 and MCF7 cell lines (Fig. 2a). We also noted considerable variation in the mutation burden of proton-treated samples between clones (Fig. 2a). Overall, this suggests that mutagenic impact of proton irradiation on SNVs, insertions and particularly deletions is evident however it also may be rather variable depending on cell-type and stochastic factors that differ between individual cells.

### Identifying SNV mutational signatures across mutagenized cell line genomes

Upon classifying SNVs into 6 different categories, tallying them DNA strand-symmetrically, i.e., C>A, C>G, C>T, T>A, T>C, T>G, the most prevalent class of SNVs in all cell lines/clones was C>A (Fig. 2b). One important cause of C>A transversion mutations in cancer genomes is oxidative damage to guanine (in Signatures SBS18 and SBS36 as reported^26^). Moreover, an abundance of C>A mutations was observed in recent mutation-accumulation experiments on human cell lines similar to ours^31,39^, plausibly due to exposure to atmospheric oxygen during cell culture conditions. Control (unirradiated) clones also had high C>A exposures.

Additionally, we observed considerable numbers of the C>T and T>C transition mutation types (Fig. 2b), which are consistent with commonly observed mutational signatures in dividing cells, namely the ‘clock-like’ mutational signatures SBS1 and SBS5/SBS40^15^. These transition mutations were particularly abundant in the MCF7 genomes, suggesting a mutational process specific to that cell line.

To further investigate mutational processes, the SNV mutations were classified into 96 categories, considering both the observed class of mutation and the trinucleotide context (A_A, A_C, …, T_T), in order to infer the 96-component mutation spectrum of SNVs for each clone (Supp. Fig. 11, Supp. Table 1).

**Figure 3:**
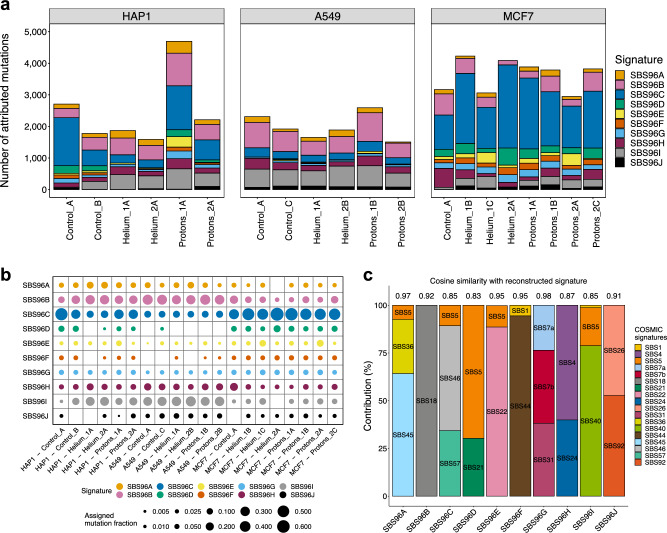
Mutational signatures of SNVs in irradiated human cell lines. (**a**) Number of point mutations attributed to each of the 10 different extracted SNV mutational signatures, for each sample in our cohort. (**b**) Exposures of all extracted SNV mutational signatures expressed as a fraction of the total mutation burden in each clone. (**c**) Decomposition of extracted signatures into PCAWG signature spectra. Values at the top of each bar represent the cosine similarity between the original signature profile and the reconstructed spectrum.

We extracted de novo mutational signatures from our irradiated and control clone WGS with the SigProfiler tool^15^. To identify similarities with known mutational processes from previous experiments on human cell line models, our data was pooled with previous genomic data: (i) 155 cell line genomes treated with various mutagens, largely chemicals, in which a specific SNV signature was able to be identified as well as gamma-irradiated genomes (with no identifiable signature)^31^, and (ii) an additional set of 38 cell line genomes generated from CRISPR-Cas9 gene knockouts of 9 DNA repair genes reported to exhibit mutagenesis upon knockout^39^. By a joint analysis of this mutation accumulation data, we identified 10 independent mutational signatures (see “Methods”) based on SNVs (here called SBS [single base substitution]) (Supp. Fig. 3a) and examined their exposure across all clones in our cohort (Fig. 3a,b, Supp. Fig. 3b,c).

In order to attribute a potential aetiology to each signature, we computed the similarity of their spectra with the catalog of known somatic signatures, here referred to as PCAWG signatures^15^ (Supp. Fig. 3d; of note, these signatures are often referred to in the literature as “COSMIC”, by the name of the database, however this is unrelated with “cosmic radiation” and this previous catalog does not contain known signatures of cosmic radiation). Additionally, we used a “decomposition” approach to model our signatures as mixtures of known PCAWG signatures (Fig. 3c). Finally, we studied aetiologies of our 10 signatures by considering the activities of these signatures in particular samples with known DNA repair deficiency or with exposures to known mutagenic chemicals or radiation from two previous studies^31,39^ (Supp. Fig. 4a).

### An overview of mutational signatures observed across cell lines

The most abundant signatures in the pooled dataset were SBS96A and SBS96B. The SBS96A signature was assigned to (Fig. 3c, Supp. Fig. 3d) both PCAWG SBS36 (a C>A signature of oxidative damage to DNA, associated with failures in base excision repair) and to SBS45 (C>A-rich signature, likely an experimental artifact due to 8-oxoG generated in vitro during preparation of DNA for sequencing^40^); the signature activities were high in the experimental exposure to certain PAHs (polycyclic aromatic hydrocarbons)^31^ (Supp. Fig. 4a). The SBS96B was assigned to the PCAWG signature SBS18, a signature resulting from damage to guanines by reactive oxygen species^15,41,42^. This signature was highly active in a *OGG1* knockout cell line genome (Supp. Fig. 4a), consistent with the known function of base excision repair protein OGG1 in mending nucleobase oxidative damage^43^ and additionally in treatments with e.g. potassium bromate (an oxidizing agent) and gamma radiation (Supp. Fig. 4a).

Next, SBS96C, a signature abundant in the MCF7 cell line genomes in our WGS dataset (Fig. 3a), was not assigned to a single reported signature in PCAWG catalog (Supp. Fig. 3d). Instead, the decomposition models it, at modest accuracy, as a mix of SBS46 (T>C transitions in various contexts, with some C>T transitions) and SBS57 (T>C and T>G mutations in TTT) (Fig. 3c). The SBS46 and SBS57 are noted to be possible sequencing artifacts in the COSMIC database. However, we infer (see below) that this is a *bona fide* mutational process not previously reported; a defect in one or more of the DNA repair pathways might generate the signature SBS96C in copious amounts in our MCF7 cells.

Signature SBS96D was not seen in previous PCAWG signatures (it is modeled, at low accuracy, as a mix of the ubiquitous, unknown aetiology SBS5, and SBS21, one signature of defective DNA mismatch repair). This signature dominated by T>C and C>T transitions, had high exposures in genomes of cell lines previously treated with methylating agents^31^ (ENU, MNU, TMZ, 1,2-DMH; Supp. Fig. 4a), suggesting that SBS96D originates from alkylating DNA damage.

The following four signatures, SBS96E–H, appear to have clear aetiologies. SBS96E corresponded closely to the PCAWG signature SBS22 (Fig. 3c, Supp. Fig. 3d), resulting from an exposure to aristolochic acid (Supp. Fig. 4a), an agent generating bulky nucleotide adducts at adenosine, resulting in T>A transversions. SBS96F was assigned to the PCAWG signature SBS44 and to the *MLH1*, *MSH2* and *MSH6* gene knockouts^39^, resembling a signature of a defective DNA mismatch repair. SBS96G was assigned to the known mutational signatures associated with ultraviolet light exposure (SBS7a, SBS7b), and had high exposure in the simulated solar (i.e. non-ionizing, UV-containing) radiation treatment of a cell line^31^ (Supplementary Fig. 4a). SBS96H is a mix of SBS4 (tobacco smoking-associated) and SBS24 (aflatoxin-associated) and was seen in cells exposed to BPDE and PhIP chemicals in previous data^31^, thus reflecting mutagenesis due to bulky adducts in DNA. These signatures, SBS96E–H, tend to generate few mutations in our samples; however, the latter, signature H, is somewhat more abundant in both treated and untreated samples (Fig. 3a,b).

Signature SBS96I likely corresponds to the ubiquitous, clock-like signatures of unknown origin SBS40 and SBS5 (although we note only an approximate spectrum reconstruction, Fig. 3c) and is abundantly present in our samples. In previous data^39^, we note a link to the knockouts of the ubiquitin ligase *RNF168* and exonuclease *EXO1* (Supp. Fig. 4a), involved in DNA damage signaling and repair respectively. Signature SBS96J contains SBS26 (DNA MMR deficiency) in its spectrum deconstruction (Fig. 3c), and it is seen in the knockouts for the *PMS2* gene of the DNA MMR pathway^39^ (Supp. Fig. 4a). However, this spectrum also contains SBS92 (tobacco smoke metabolites exposure) and is found in genomes treated with 6-nitrochrysene; thus, its underlying mechanism is unclear, or it may represent a mixture of mechanisms.

### Widespread SNV mutational signatures that are differentially active between cell lines

We further considered signature activity in a particular sample in relation to the treatment and/or to the cell line of origin. In order to systematically compare between these groups, we implemented a randomisation test strategy (Supp. Fig. 5a, see “Methods” for details).

Firstly, we consider the three most abundant signatures in our cell line data: SBS96B (oxidative DNA damage, ~ SBS18), C (possible DNA repair defect and/or artifacts), and I (background mutagenesis, ~ SBS5/40). These were found at various levels in the three cell lines and in the majority of the samples of each line and were also seen in the non-irradiated control samples (Fig. 3b).

The canonical signature of reactive oxygen damage, SBS96B, was detected also in the non-irradiated clones, probably resulting from DNA oxidation reactions during cell culture. SBS96B, however, does trend towards a higher activity (Supp. Fig. 5a; p = 0.079; n.s. upon FDR adjustment) in the proton irradiated versus helium irradiated cell lines (Fig. 3b; Supp. Fig. 5a). This trend is seen when considering the cell lines jointly, and also when considered individually in comparisons of proton *versus* helium treatments (Supp. Fig. 5a). Our data is consistent with a mechanism of more rapid formation of reactive oxygen species as a consequence of proton radiation, however it could also result from other mechanisms in principle (hypothetically, a higher rate of DNA damage resulting from the reactive oxygen, and/or compromised repair of oxidized DNA upon proton radiation). Irrespective of the treatment, this DNA oxidation signature was seen at different abundance across the cell lines, in order A549>HAP1>MCF7.

Our SBS96C signature, modeled as a mixture of two known PCAWG artifact signatures, is present differentially in all MCF7 cell line WGS versus the other two cell lines—A549 and HAP1—where it is rare (Supp. Fig. 5a, p = 0.00008 and p = 0.185, respectively). SBS96C does not associate with radiation exposures. Since all our samples and resulting data were treated equally, it does not seem likely that a sequencing/alignment/calling artifact would arise in data from one cell line, while largely absent in the other two. Additionally, the reconstruction of SBS96C as a mixture of the artifact signatures SBS46 and SBS57 was not highly accurate (Fig. 3c, cosine similarity = 0.854), further supporting that this is a genuine mutagenic process, possibly an acquired DNA repair deficiency. SBS96D was found in abundance in all samples of the MCF7 cell line including the control, and interestingly also in two clones from the HAP1 cell line including one control. We infer this may be a form of MMR deficiency, based on the co-occurrence with indel signatures (see below), possibly combined with other DNA repair deficiencies thus explaining the unique SBS spectrum not observed in previous cell line data considered here.

Another abundant signature was SBS96I, referring to a SBS40-like background mutagenesis. SBS96I was not associated with particle radiation treatments in the 3 cell lines considered jointly, however its abundance was strongly variable across cell lines in the order of A549>HAP1>MCF7 (Fig. 3a,b, Supp. Fig. 5a), consistently as SBS96B above. This potentially indicates a clock-like nature of signatures SBS96I and SBS96B in our experimental setup, inferred from a slow division rate of MCF7 cells (not shown).

### Further signatures of DNA damage and deficient DNA repair not associated with particle radiation treatments

SBS96F, the DNA repair deficiency signature that strongly associated with deletion of the MMR genes *MLH1*, *MSH2* and *MSH6* (Supp. Fig. 4a) was not highly active in our samples; instead, it was seen mostly in the external datasets we integrated. Consistently, no association with either type of radiation treatment was observed in any of the 3 cell lines (Supp. Fig. 5a). Thus, particle radiation exposure does not commonly select for cells with a deficient MMR system, unlike other types of chemical exposures which can generate mismatch-like DNA lesions^44^. In accord with this, SBS96J, which may also be associated with DNA repair inefficiency via ablation of the *PMS2* mismatch repair gene (Supp. Fig. 4a), is overall present at low levels, but more commonly in A549 and MCF7 compared to HAP1 (Fig. 3a,b) and again, appears to not be associated with treatment in 2 cell lines (some association noted in MCF7 cells only; Supp. Fig. 5a). Overall, mutational signatures of deficient MMR in our data appear unrelated with alpha or proton radiation exposure, however can differ in activity between cell lines.

The SBS96D signature, likely originating from methylating DNA damage, is not associated with particle radiation treatment, but differs considerably between cell lines in activity (MCF7>HAP1>A549; Supp. Fig. 5a). Similarly, the SBS96A signature, likely resulting from exposure to certain types of oxidative DNA damage and/or failures to repair the damage, does not show association to our particle radiation treatment overall nor in 2 of the cell lines (there may be some signal in HAP1 cells; Supp. Fig. 5a).

The mutational signature of the non-ionizing UV radiation SBS96G, was, expectedly, detected at only minor levels (≤ ~ 5% of observed mutations) in our data (Fig. 3a,b). While overall it was slightly (albeit positively) associated with proton radiation treatment (Supp. Fig. 5a, p = 0.075 for control vs protons; n.s. after FDR adjustment), this association was seen only in one cell line.

### Three SNV mutational signatures with tentative links to particle radiation treatments

In addition to the common C>A signature of oxidative stress SBS96B mentioned above, we noted some evidence for radiation treatment association in our cell line experiments with two other signatures. Firstly, the T>A transversion signature SBS96E (Fig. 3a,b), associated with bulky DNA adduct-forming mutagen exposures: while present overall at low levels in our data (somewhat higher in MCF7 cells) was modestly positively associated with proton treatment (Supp. Fig. 5a, p = 0.097 for control vs protons). Similarly, another signature associated with bulky DNA adducts, the SBS96H (seen in previous BPDE-treated samples and similar to tobacco-smoking SBS4), was consistently trending towards positive association with proton treatment compared to helium treatment (Supp. Fig. 5a, p = 0.160 for helium vs protons; n.s. after FDR adjustment). For the SBS96E and SBS96H associations with particle radiation, these trends were seen in at least two out of three cell lines and are thus plausible, however it should be noted that the effects were modest and did vary across the cell lines. One possible explanation is that there is genuine variation between cell types and/or genetic background in the mutational signatures of particle radiation. However, it is also possible that the subtle effect of particle radiation on SNV spectra is in fact universal but did not cross a detection threshold in one of the cell lines.

The mechanisms that underlie signatures SBS96B, E and H have a connecting thread: these mutational signatures are thought to result from large, replication-blocking DNA lesions. Thus, it appears that proton/helium radiation effect on DNA can mimic the mutational footprint of mutagens generating bulky adducts. Speculatively, such a DNA lesion might be generated by the radiation causing intrastrand nucleotide cross-links, or by cross-linking DNA with proteins. Since these SNV mutational signatures were found in both protons and alpha radiation treatment groups (of note, more so in the former), they do appear broadly relevant to various particle radiation types present in GCR. Moreover, they would be expected to result from other sources of particle radiation (e.g., in cancer radiotherapy using protons) in the susceptible cell types and/or genetic backgrounds.

As a technical check, based on repeated NMF runs on subsampled WGS data, we demonstrated that including a large amount of data from two prior studies^31,39^ in our signature extraction dataset, as described above, does not have undue influence on the NMF point mutation signatures identified here (see “Methods” for description; Supp. Fig. 6a,b).

### Mutational signatures of indels across cell types and conditions

Indels can be classified into 83 types (the ID83 categorization, based on PCAWG indel signatures^15^). In brief, the indels are separated into 1-bp or longer indels, insertions or deletions, at DNA repeats or not, and finally deletions with micro-homology are counted separately. These classes can further be divided into subclasses based on the gained/lost nucleotide or based on the alteration length. Each observed indel is assigned to one category, thus providing the mutational spectrum of indels for each cell line and each condition (Supp. Fig. 12, Supp. Table 1).

To estimate the indel mutational signatures, of which some are potential footprints of the helium and alpha radiation treatments, we ran the SigProfilerExtractorR tool. The indel count matrices were derived from the same pooled dataset, consisting, as for SNVs discussed above, of the genomes from our irradiated MCF7, A549 and HAP1 cell lines, as well as genomes of mutagen-treated and DNA repair deficient cell lines from previous studies^31,39^ (see “Methods” for details).

**Figure 4:**
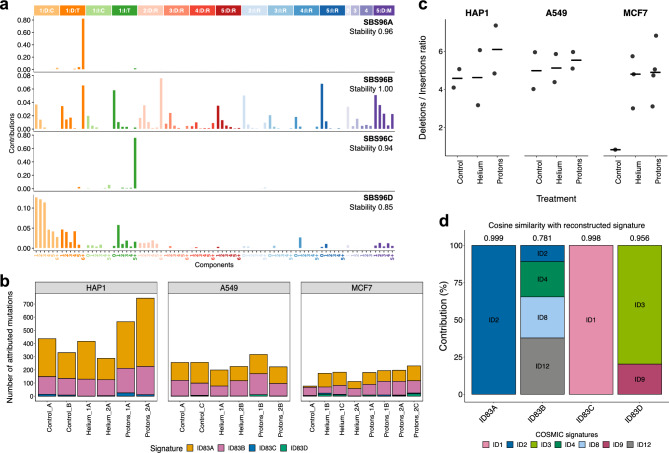
Mutational signatures of small insertions and deletions in irradiated human cell lines. (**a**) Mutational profiles of the 4 indel signatures extracted using the SigProfiler ExtractorR algorithm. Indels were classified into 83 distinct categories following the procedure described in Alexandrov et al*.*^15^. (**b**) The number of indels attributed to each of the 4 different signatures, for each sample in our cohort. (**c**) Ratio of all small deletions over all small insertions, pooled by treatment for each cell line. Black horizontal lines denote the median of each group. (**d**) Decomposition of extracted signatures into PCAWG indel signature spectra. Values at the top of each bar represent the cosine similarity between the original signature profile and the reconstructed spectrum.

Four indel signatures were identified: ID83A, ID83B, ID83C and ID83D (Fig. 4a), as well as the activity (“exposure”) of each in our experimental cell line samples (Fig. 4b). We also compared simply the burden of small insertions and deletions in each treatment group, for each cell line (Fig. 4c). To infer potential underlying mechanisms, we also examined the estimates of the signatures’ activity in previous DNA repair knockout and mutagen-treated cells^31,39^ from which signatures were extracted (Supp. Fig. 4b), as for the SNV signatures above. ID83A, consisting of 1 nt deletions at A:T homopolymers closely matched the PCAWG ID2 signature (Fig. 4d, Supp. Fig. 3e) and was abundant in DNA mismatch repair deficiency (*MSH2*, *MLH1* and *MSH6* knock-outs) (Supp. Fig. 4b). Its sister signature, ID83C, consisting mainly of 1nt insertions at A:T homopolymers, matched the PCAWG ID1 (Fig. 4d, Supp. Fig. 3e), suggesting a link to polymerase slippage during DNA replication^15^. While ID83A contributes highly to the mutation burden of our samples, ID83C is responsible for a small number of mutations in HAP1 samples. Regarding the association to treatment, while the ID83A and C did display a weak positive association for protons, this was mainly seen in one cell line (HAP1 in both cases) so ID83A and ID83C do not appear to be a general signature of particle radiation exposure. The activities of the indel signatures correlated with activities of some SNV signatures across our cohort (Supp. Fig. 7), suggesting possible mechanistic links. In particular, ID83C activity is significantly positively correlated with SBS96D exposure, further supporting the idea that these signatures represent features of MMR deficiency.

The remaining 2 signatures, ID83B and ID83D, were more interesting. ID83B was modeled as a mixture of 4 PCAWG signatures (Fig. 4d), with higher weights of the ID12 (unknown aetiology) and ID8 (ionizing-radiation associated signature)^30,45^ and ID4 (unknown aetiology). The spectrum of ID83B consists of various lengths (1–5 bp with similar frequency) insertions and deletions outside of DNA repeats, as well as a notable component of deletions with short flanking microhomology (mostly 1–2 nt; we note this contrasts with the microhomology-flanked deletions resulting from homologous recombination deficiencies, which tend to be ≥ 2 nt^46^). The activities of ID83B in prior cell line WGS data correspond to exposures to UV radiation and to propylene oxide and to furan (Supp. Fig. 4b). In our experiments, the ID83B signature was overall abundant and modestly enriched in proton-treated samples when considering the 3 pooled cell lines (Supp. Fig. 5b, compared to helium-treated, p = 0.017; there was a weaker trend when compared to untreated, p = 0.18). consistent direction of effect was observed in all 3 individual cell lines (Supp. Fig. 5b) supporting a link of ID83B with particle radiation exposure.

Secondly, the ID83D signature consisted mostly of short 1-bp and 2-bp deletions outside of homopolymeric repeats, plus an additional minor component of ≥ 5 bp deletions with short flanking micro-homology. ID83D was found to be active in a cell line with *RNF168* knockout^39^, a gene encoding a DNA damage signaling protein (Supp. Fig. 4b), and in cells treated with PAHs from tobacco smoke (BPDE, DBADE and B[a]P, Supp. Fig. 4b); consistently, the signature contained a component of PCAWG ID3 (Fig. 4d), found in tumors of tobacco smokers. This signature displayed a weak association with proton and helium exposures, seen in two cell lines (Supp. Fig. 5b).

Consistent with trends toward association with radiation treatments in our study, certain features of ID83B and ID83D spectra are similar to the indel mutational signature induced by ionizing photon radiation in clonal organoids from mouse and human cells^45^. Overall, because the association of ID83B with radiation does not appear specific to cell lines (i.e., there are no tissue-specific nor genetic background-specific effects), this suggests a universal indel mutational footprint of small deletions caused by particle-based radiation in human cells. However, due to a small number of cell lines studied herein, we do not rule out that different cell types and/or genetic background might be differentially affected by different radiation types generating indel changes.

### Mutational spectra of structural variation in irradiated cells

**Figure 5:**
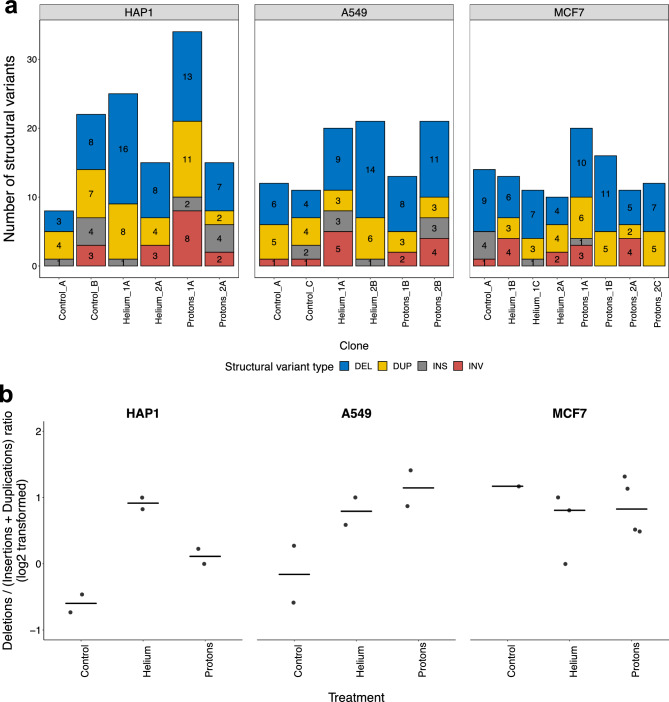
Structural variation in irradiated human cell lines. (**a**) Distribution of the number of structural variants (SVs), decomposed into large deletions, large insertions, duplications and inversions, per clone in each of the three analyzed cell lines. (**b**) Ratio of all large deletions divided by all large insertions and duplications, pooled by treatment for each cell line individually, log2 transformed. Black horizontal lines denote the median of each group.

Large structural variants (SVs, also referred to as rearrangements) were grouped into four broad categories: large deletion (DEL), large insertions (INS), tandem duplications (DUP) and inversions (INV). No particular SV category has been found to be the most prevalent across the cell lines and across the cell lines/conditions (Fig. 5a, Supp. Fig. 2).

The rarest event type—insertions—is similarly observed across the cell lines and clones; their low frequency may however stem from technical difficulties in calling insertion variants from short-read WGS, resulting in many undetected variants. More generally, in our experiments, we observed globally a low number of SVs of any type, suggesting that the impact of the proton and alpha particle treatment on rearrangement rates in human cells may not be remarkable, at least in the treatment regimen applied here. The helium and proton-treated clones from all cell lines have higher numbers of large deletion SVs compared to the untreated clones (Supp. Fig. 2), although when SVs are considered in summary, the irradiated clones overall are not strongly enriched compared to nonirradiated ones. We also note a substantial variability between individual clones in SV burden.

We also investigated another quantitative measure of the identified structural variation: the distributions of SV sizes, as commonly done in cancer genome analyses^27,29^. Again, we asked if there is a difference between two types of radiation treatments, also comparing them with the non-irradiated controls. Because insertions were rarely detected, here we considered only deletions, duplications and inversions. While the distribution of SV lengths was largely not substantially different across the conditions, nevertheless, we observed some cell line specific trends. In HAP1, duplications are smallest in unirradiated clones and largest in proton treated clones, with a significant difference in length between the two (Supp. Fig. 8, p = 0.046). In A549 clones, on the other hand, the opposite is true: duplications in untreated clones are longest and significantly different from helium and proton clones (Supp. Fig. 8, p = 0.04 and p = 0.026 respectively). Cell line specific effects are a possibility, but so are technical challenges with calling SV from short-read sequencing data.

Inversions appear not strikingly different in size between all cell lines and treatment groups (Supp. Fig. 8), however the sample sizes are too small to ascertain significance of any trends. In case of deletions, we observed a trend that is consistent across all the cell lines. In particular, proton radiation generated the shortest deletion events, followed by alpha particles, and finally the longest deletions were observed in untreated controls (Supp. Fig. 8). Inversions behaved similarly: treated clones, whatever the radiation type, tended to contain inversions smaller than in non-treated samples. Moreover, there was a trend toward longer inversions resulting from helium ions compared to protons (Supp. Fig. 8). Overall, proton radiation generated SVs of shorter lengths compared to alpha particles, or compared to the SVs generated during a baseline mutagenesis in untreated cells. This suggests a different mode of DNA damage and/or repair thereof following proton exposure in various human cell types.

Finally, we investigated the balance of burden of deletion SVs versus insertion and duplication SVs (the latter two were merged for this analysis). As reported for IR-associated second malignancies^14^, the ratio of deletions to insertions increases in our particle-irradiated human cell lines when pooled together, and the trend stems from HAP1 and A549 clones (Fig. 5b, Supp. Fig. 2). Overall, different types of IR, either X-rays or gamma as employed for cancer therapy^13,14^, or protons and alpha particles as tested here, exhibit an overall similar spectrum of increase in deletion SVs relative to insertion/duplication SVs. When drawing parallels between this SV analysis to the indel analysis, considering the deletion to insertion ratio, we observed a similar trend towards more deletions in proton-treated clones compared to untreated (Supp. Fig. 2, p = 0.055 for small indels, p = 0.082 for SVs). For large SV insertions and deletions, we observed a higher ratio also in helium treated clones compared to control (Supp. Fig. 2, unadjusted p = 0.014), a trend which retains the direction (but does not reach significance) in the small indel ratio of deletions to insertions. In summary, a relative increase in deletion to insertion mutations results from exposure to particle radiation in human cells.

### Regional mutation rates with respect to replication time and gene activity

DNA replication timing (RT) is correlated with regional variability in somatic mutation rates in human tissues. There is an increased mutation rate in late-replicating heterochromatic regions of the genome, because DNA repair mechanisms preferentially protect the early-replicating euchromatic, gene-rich regions^47,48^. We were interested to know if there is an enrichment of radiation-associated mutation rates in regions with a specific RT.

Overall SNV mutation rates were, as expected, associated with RT in all 3 cell lines (Supp. Fig. 9), with reduced rates in early-replicating domains. The SVs observed did not display a significant association but weakly trended towards the converse association (Supp. Fig. 9); we note the modest number of SVs makes it difficult to detect associations. However, with respect to radiation treatment, we did not observe a significant change of correlation between SNV density and RT bins, or SV density and RT bins, between treatments in any cell line (Supp. Fig. 9). This suggests that exposure to particle radiation does not impact the large-scale distribution of mutation risk along the RT domains in the human genome.

Next, we considered differences in mutation rates at the gene scale by investigating the distribution of SNV and SV mutations across regions harboring various levels of gene expression (Supp. Fig. 9). As expected, higher gene expression was associated with lower SNV rates; we note a nonsignificant opposite trend for the SVs (Supp. Fig. 9). However, there was no consistent effect of radiation treatment on the association between gene expression and SNV or SV burden across the three cell lines (we note some effect in SNV mutations that appears particular to the MCF7 cell line, and so tissue-specific effects or genetic background-specific effects cannot be ruled out). Thus, a particle radiation treatment does not preferentially increase or decrease relative mutation rates in transcriptionally active regions of the genome.

### Clustered mutation processes due to particle radiation exposures

**Figure 6:**
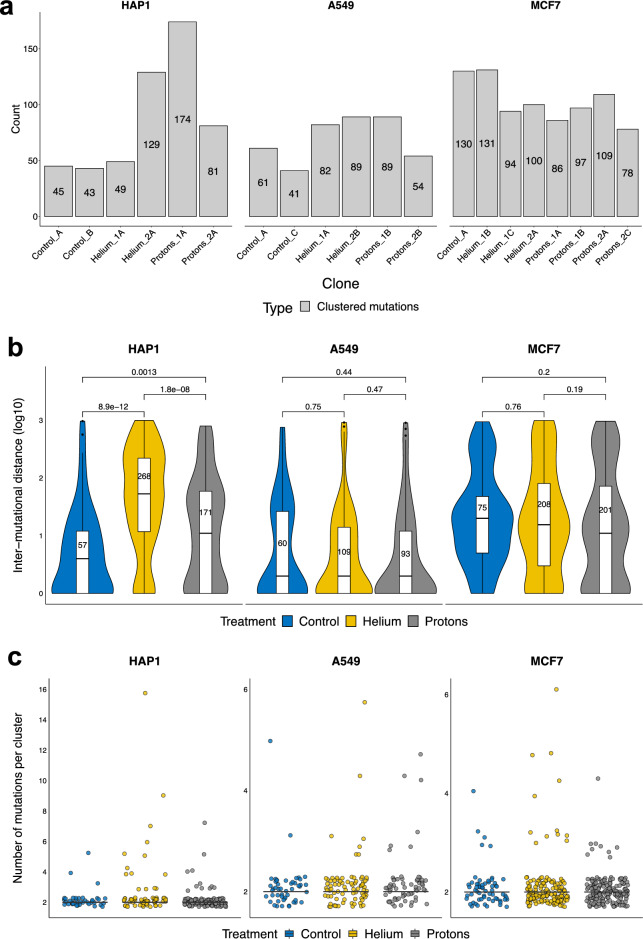
Clustered point mutations associated with irradiation. (**a**) Number of total clustered mutations per clone in our cohort. (**b**) Distribution of the log10-transformed genomic distance between point mutations that were identified as being clustered together (see “Methods” for the definition of a mutational cluster); note the distance between two unique mutations is only counted once. The number assigned to each treatment denotes the total number of cluster inter-mutational distances plotted, whilst the black horizontal line represents the median inter-mutation distance. *P*-values shown were computed using the *ggpubr* R package v0.4.0 (*t* test mean comparison of distances). (**c**) Distribution of point mutation cluster sizes, pooled by treatment for each cell line individually.

Next, we considered patterns of mutation clusters, which can be particularly informative about various mutational processes^28^, most prominently with regard to APOBEC enzyme DNA damaging activity^49–51^ and also error-prone DNA polymerase usage^28^. Operationally, we defined a mutation cluster as a set of point mutations at a pairwise inter-mutational distance lower than 1 kb; given the overall modest mutation burden in our genomes, this threshold is unlikely to result in artefactual (false-positive) mutation clusters. In the A549 cell line, 102 mutations were clustered in control clones, 171 in helium-irradiated clones and 143 in proton-irradiated samples. In the HAP1 cell lines, a total of 88 mutations were clustered in control clones, 178 in helium-irradiated clones and 255 in proton-irradiated samples. Finally, in the MCF7 cell line, we detected 130 mutations in the control clone, 325 clustered mutations in the helium-irradiated clones and 370 in the proton-irradiated clones (Fig. 6a).

We detected more clustered mutations in radiation conditions compared to non-radiation conditions (control) in all cell lines, indicating that alpha and proton radiation generates mutation clusters in various human cell types (Fig. 6a, Supp. Fig. 10a). This pattern of mutagenesis is consistent with many reports of IR damaging DNA in a clustered manner^52–54^. With respect to types of radiation, more clustered mutations are found in helium clones compared to proton clones in all cell lines, and the trend is particularly salient in HAP1 clones (Fig. 6a).

We next asked if the clustered mutations, depending on the cell line and the radiation type, were found at closer or longer intermutational distances. In HAP1 cells, clustered mutations were spaced significantly further apart for both helium and proton radiations compared to the control clones, with the helium group displaying the largest inter-mutational distance (Fig. 6b). The trend is however not clearly seen in the other cell lines, despite differences in mutation counts (Fig. 6b).

We were interested in the arrangement of the clustered mutations, i.e., do they form a lot of small clusters or do they form a few large (multi-mutation) clusters, the latter case corresponding to a *kataegis*-like phenomenon^49^ and the former to an *omikli*-like phenomenon^51^. We applied a graph-theory approach to quantify this (see “Methods”). Considering large clusters as connected components of size higher than 5, we identified one large cluster in A549/helium, four in HAP1/helium, one in MCF7/helium and one in HAP1/proton. However, there were no large clusters in the untreated cells, suggesting mutation showers can be generated by IR (Fig. 6c). Of note, the majority of clustered mutations, both in the irradiated and in the untreated conditions, were in smaller, *omikli*-like clusters; for most cell lines and conditions, over 90% of clusters consisted of two mutations (Fig. 6c).

Finally, we were interested in the mutational spectrum of the clustered SNV mutations, which can identify or rule out a mechanism underlying the clusters. Here, because of the relatively modest number of SNVs, we considered the 6 mutation type-spectrum of clusters (Supp. Fig. 10b) and compared it with the spectrum for all SNVs (Fig. 2b). The clustered SNV spectrum (Supp. Fig. 10b,c) differed from the general SNV spectrum in that clusters had a substantially lower proportion of C>A changes (largely due to oxidative stress during cell culture), and a somewhat lower proportion of T>C transitions (which can result from multiple causes). The proportion of C>G and T>G changes is similar between the clustered and unclustered. C>T and T>A changes are slightly more prevalent in clustered mutations compared to the total, although variation between clones exist. The lack of high C>G enrichment in clustered mutations, as well as the lack of TCN>G/T mutations in the 96-class spectrum (Supp. Fig. 13, Supp. Table 2) suggests that the activity of the APOBEC3 cytidine deaminase is likely not responsible for the clustered mutation burden in our experiments.

Overall, this suggests that the clustering of mutations in human cells often results from exposure to particle radiation, that the underlying mechanism is unlikely to be APOBEC-related, that a process with a similar mutation spectrum may be active (albeit at much lower levels) also in unirradiated cells, and that radiation may occasionally generate multi event, *kataegis*-like mutation clusters.

## Discussion

In this study, we analyzed the genomes of three human cancer cell lines irradiated with two different components of GCR—proton and helium exposure—in order to characterize the spectra of DNA alterations that GCR would produce in different human tissues. Overall burden of SNV mutations, indels and SV events was not markedly different between treated and untreated cells. This suggests that particle radiation exposure—at least in the regime applied in our experiments—is not grossly mutagenic (see below for further considerations).

However, there were certain differences in mutation spectra and distributions between particle beam treated and untreated cells. For instance, in agreement with a recent study analyzing IR-associated tumors^14^, for indels we observed an enrichment of deletions compared to insertions, and this was even more the case for large (SV) events (Figs. 4c, 5b). This phenomenon was also observed in a recent study that reported a 3.6-fold increase of small deletion burden when comparing radiotherapy-treated glioma patients to untreated glioma patients^13^. We note that tumor radiotherapy usually employs X-rays or gamma rays, rather than the protons and alpha particles tested here, and so this enrichment of deletion mutations appears broadly independent of the type of IR applied to cells.

We found that particle radiation generates point mutations that are more often clustered. This is in line with recent work suggesting clustered mutations to be more specific indicators of certain types of mutagenic processes in human^28,55,56^ than the general, genome-wide mutation signatures. The SNV spectra of the clustered mutational distributions generated by helium and proton radiation exposure do not indicate a major role of the known agents commonly generating clustered mutations in human cells: the APOBEC3A enzyme and the translesion synthesis DNA polymerase eta (POLH)^28,55^. Mechanisms would need to be clarified with additional experiments in future work.

We were also interested in the SNV trinucleotide mutational signatures that helium and proton exposures can generate, and whether known biological mechanisms can be linked to those footprints. While most observed SNV signatures were consistent across all treated and untreated samples, e.g., reactive oxygen species mutagenesis was noted universally, some samples exhibited signatures consistent with MMR deficiency. This MMR deficiency was however unlikely to have resulted from, or have been selected by irradiation, because we detected it also in a control sample. Interestingly, we observed a modest enrichment of certain SNV signatures in both irradiation conditions: a C>A signature linked with oxidative stress, and an T>A rich signature, resembling previous signatures of aristolochic acid and some chemotherapy treatments^49,50^, suggesting, speculatively, that particle radiation may generate lesions resembling bulky adducts.

Similar to SNVs, some indel signatures were radiation-associated, being consistently enriched (with modest effects) exposed samples, thus suggesting a likely general effect over tissues for indel-generating mechanisms. The indel signatures bore certain similarities with indel signatures previously reported for photon IR^30,45^; additional data appears required to gain statistical power to establish to what extent the indel signatures indeed differ between photons and particle radiations, providing more insight into mechanisms.

Regarding non-ionizing radiation, the solar radiation-generated SNV signatures identified previously^15,31^ (presumably generated by the UV radiation) were observed at low intensities in our irradiated samples, likely stemming from imprecisions during statistical analysis. This is consistent with prior knowledge that ionizing cosmic radiation affects the DNA differently than the non-ionizing UV radiation^56^.

Our study also has several limitations. For instance, some components of GCR were not considered in this study, in particular the heavy atom nuclei (often referred to as “HZE ions”), highly energetic particles likely originating from supernova explosions. Despite their small proportion in cosmic radiation, their biological impact might be large, and their mutagenic effects on genome stability remain to be studied.

Next, we considered only a single dose of radiation that resulted in a ~ 40% to 50% reduction of cell viability assessed by the colony formation assay, and a fractionation regime of four total fractions (with each fraction given every 4 days) exposures of this dose. The dose studied here had rather strong biological effects on cell viability and does not correspond to what would be encountered in space travel (where any vessel would need to be shielded to prevent such exposures that would have very deleterious acute effects on the organism). These exposures were chosen as an experimental setup, with the rationale that doses with notable effects on cell viability would probably be well sufficient to observe the mutagenic effects of particle radiation, if any. Since a gross increase in mutation rates was not observed, we infer that particle beam exposures are not highly mutagenic. However, we cannot rule out the (less parsimonious) scenario where lower doses of particle radiation than those employed here might be more mutagenic e.g., by failing to trigger cell cycle checkpoints, thus ‘slipping under the radar’ of the mechanisms protecting genome integrity and introducing mutations.

Moreover, given that for various mutation patterns we observed responses that appear specific to one cell line, this suggests there may be tissue-specific and/or genetic background specific responses (these two scenarios cannot be distinguished from our data). Additional experiments on different cell lines or other experimental models would be required to ascertain tissue-specific responses to various radiation types. Furthermore, this study was performed on cancer cell lines, and mutational responses might be different in healthy, noncancerous cells, which remains to be investigated in future work.

In conclusion, our study suggests that particle radiation components of the galactic cosmic radiation are not overly mutagenic to human cells, with very modest effects on point mutation spectra, and with some effects on SV distributions, indels and on clustered mutation spectra. However, the repeated exposure regime that we employed is different from longer-term, chronic exposures to GCR expected e.g. during space flight. Even modest increases in mutation rates under chronic GCR exposures might have detrimental effects on cancer risk, and possibly neurodegeneration and reproductive health and on genetic disease incidence in the progeny. Therefore, we highlight the necessity of further experimental work on cell and animal models, using longer-term exposures to various components of galactic cosmic radiation to characterize their effects on the stability of the genome.

## Methods

### Cell lines

We used three human cell lines: A549, HAP1 and MCF7. The lung adenocarcinoma cell line A549 (CCL-185) and the breast adenocarcinoma cell line MCF7 (HTB-22) were purchased from ATCC (the American Type Culture Collection, Manassas, VA). The near-haploid cell line derived from the KBM-7 cell line HAP1 (C859) was purchased from Horizon (Carle Place, NY). All cell lines were cultured and maintained according to recommended protocols. All the cell lines were authenticated by STR profiling by the corresponding repositories.

All cells were grown at 37 °C in a 5% CO_2_ humidified incubator; the A549 cells were grown in Eagle’s Minimum Essential Medium (MEM), MCF7 in Dulbecco modified MEM, and HAP-1 cells in Iscove’s modified Dulbecco's medium (IMDM). All the media were supplemented with 10% heat-inactivated (56 °C, 30 min) fetal bovine serum (FBS), and 100 U/ml penicillin and 100 mg/ml streptomycin (all from Sigma-Aldrich Corp).

### Cell irradiation, culture and clone isolation

A clonogenic assay^57^ pilot experiment was conducted to assess the dose that resulted in 40–50% lethality (i.e., 50–60% survival) to 11 MeV helium ions (LET = 65 keV/µm) or 5.4 MeV protons (LET = 10 keV/µm), which was 0.5 and 1 Gy, respectively (Fig. 1a,b). Cells for the experiment were then exposed to four total fractions delivered every 4 days. Cells were collected 4 days after the last fraction. For each sample, half of the cells were frozen at − 80 °C while the other half was re-plated until confluency. The cells were then collected, separated by vigorously passing through a 21 G syringe and 3 μl/ml of DAPI was added to the solution for cell sorting at the BD Influx cell sorter. For each cell line, single cells were sorted in two 96-well plates and incubated in 250 μl of medium supplemented with 20% FBS. Once colonies were visible, we picked ~ 30 colonies and re-plated each one in an individual well of a 12-well plate in the case of A549 and MCF7 and in 48-well plate in the case of HAP1 cells up to confluence. The cells were then collected and half were frozen at − 80 °C while the other half was replated in 6-well plates and then in 100-mm Petri dish until confluence. DNA was extracted with PureLink Genomic DNA kit (Invitrogen), frozen and shipped to the sequencing centre.

### Cell irradiations at the track segment irradiation platform

The 5.5 MV Singletron accelerator at the Radiological Research Accelerator Facility (RARAF) served as a source of energetic protons and alpha particles. The accelerator was operated at the maximum terminal voltage generating beams with nominal energies of 5.5 MeV and 11 MeV for protons and alphas, respectively. Cells were irradiated at the so called “track segment” irradiation platform whose name indicates the traversal of thin samples (typically cell monolayers) by a short segment of the ion’s trajectory resulting in a small variation of the linear energy transfer (LET) throughout the sample. It is valid therefore to assume that radiation doses at the track segment platform are delivered by mono-LET beams. The LET values of the applied beams were 10 keV/µm for protons and 65 keV/µm for alphas. Detailed description of the track segment irradiation platform and its operational principles can be found elsewhere^1–4^. We will state here only the main features of the irradiation protocol. The protocol can be generally divided in two parts. First, beam characterization and dosimetry were performed with the use of different detectors mounted on the metal wheel that rotates over the beam exit aperture. The aims of this step were to verify the energy and the LET of the beam, to check the uniformity of the irradiation field covering the 6 mm × 20 mm beam exit aperture (2.9 µm thick Havar foil) and, finally, to calibrate the online beam monitor in terms of the absorbed dose delivered to the sample. In the second step, the dosimetry wheel was replaced with the sample-carrying wheel that can accommodate up to 20 custom made dishes. The dishes were manufactured by gluing 6 µm thick mylar foil over metal rings having a diameter of 5 cm. The mylar foil serves as the bottom of the dish on which the cells are attached, allowing thus the ions to penetrate through and reach the cells without losing much of their energy. The in-house developed software drives the stepper motor and controls the movement of sample dishes over the beam. A desired dose was delivered to the sample by exposing it to the beam until the appropriate number of monitor counts was reached according to dosimetry measurements performed during the first part of the irradiation protocol.

### Irradiations of the cells for mutational analysis and clone isolations

The obtained survival curves were fitted with the linear quadratic (LQ) model. Doses of 1 Gy and 0.5 Gy were selected for investigating the mutational signatures of protons and alpha particles, respectively. According to the LQ fits, the selected doses resulted in preservation of between 50 and 60% clonogenic capacity for all cell lines. Four fractions of the same dose were delivered to the cells every 96 h to amplify the number of mutations in surviving cells. Between the fractions, the cells were not removed from the mylar dishes. To prevent the cells from overgrowing the dish size before the end of the irradiations, the initial number of plated cells was < 1000.

### Whole-genome sequencing

Total genomic DNA was extracted from pelleted cells using PureLink™ Genomic DNA Mini Kit (K182001, Thermo Fisher Scientific). DNA was sequenced on NovaSeq 6000, 150 nt paired-end mode. Reads were aligned to the reference human genome Hg38 (GRCh38.d1.vd1) using BWA v0.7.17^58^. GATK Base Quality Score Recalibration was applied to adjust quality scores assigned during the sequencing process^59^. The average coverage of the various samples was 18.5X to 44.8X.

### Variant detection

Strelka2 variant calling algorithm v2.9.10 was used to detect point mutations and small insertions and deletions^60^. Strelka2 was launched in the joint genotyping mode: briefly, mutations were called for each clone individually (in one batch), but genotyped jointly across all clones belonging to a given cell line. Mutations were then annotated using annovar, function table_annovar.pl^61^ with the gnomAD genome database v2.1.1 allele frequencies^62^. This allowed us to identify mutations shared by all clones (which were acquired prior to treatment) as well as discrete mutations acquired by only one clone during treatment. To arrive at the final set of mutations used in our analyses, a number of filters were applied: (1) variants passed both sample and call filters from Strelka2, (2) variants were found in uniquely mapping region of the genome (based on the Umap k50 mappability tracks), (3) variants were either not present in the gnomAD database or found at frequencies lower than 0.1%, (4) variants were genotyped in only one of the cell line clones, (5) variants were not multiallelic and (6) there were 2 or more variant supporting reads in the genotyped clone. For small insertions and deletions, we also imposed a genotype quality equal or higher than 10 and removed variants where more than two other clones have one or more variant supporting reads and variants where more than one other clone has two or more supporting reads, in order to remove putatively germline mutations only genotyped in one clone by Strelka2.

To call structural variants, i.e., large insertions and deletions, duplications and inversions, we used the Manta v1.6.0 tool in the joint calling mode^63^, in the same manner we used Strelka2. For inversions, Manta returns a list of breakpoints that describe the inversions it detected. To transform those breakpoints into inversions, we applied the convertInversion.py python script provided by the Manta developers (https://github.com/Illumina/manta). Filters 1–5 described above were also used to filter the structural variant calls; further, we required variants to have 2 or more supporting split-reads in the genotyped clone, with no requirement for the number of supporting spanning reads.

### Mutation clustering

Point mutations were considered to be clustered if they appeared at positions with a genomic distance lower than 1 kb; such mutations would be classified as *omikli* (“mutation fog”)^51^. To consider large, multi-mutation clusters resembling a *kataegis* (mutation shower) event^49,64^, we used an approach based on graph theory. Roughly, we consider each mutation to be a node of the graph, and edges connect two nodes if the distance between corresponding mutations is lower than 1 kb. Then, we computed the components of the graph, that corresponds to a connected sub-graph, and subsequently the size of the components, i.e., how many nodes or mutations are present in the component.

### Extraction of mutational signatures

We pooled our samples (21 clones) with two already published datasets in order to help NMF converge and in order to compare our datasets to signatures of DNA damage and of various mutagens. We first pooled our data with the mutations reported in the Zou et al*.* study^39^, which analysed 38 clones generated by CRISPR-Cas9 knockouts of 9 DNA repair/replicative pathway genes (Δ*OGG1*, Δ*UNG*, Δ*EXO1*, Δ*RNF168*, Δ*MLH1*, Δ*MSH2*, Δ*MSH6*, Δ*PMS1*, and Δ*PMS2*) which generated mutational signatures in human induced pluripotent stem cells (note that there were additional gene knockouts which did not produce mutational signatures). We then pooled the data with the mutations reported in the Kucab et al*.* study^31^, which reported 153 clones (treated with 53 environmental agents) yielding a mutational signature (note there were additional samples in the study without a prominent mutation pattern, which were not included here), as well as 2 clones treated with gamma radiation (with no identified signature).This pooled dataset of 214 individual clones was used for SNV and indel signature extraction. In order to extract SNV signatures generated by our radiation treatments, we applied SigProfiler ExtractorR v1.1.16 algorithm^15^ with default settings on the set of detected mutations, classified into the 96 SNV categories. The suggested solution contained 10 extracted signatures with high stability and low reconstruction error. Extracted signature spectra were compared and assigned to a known PCAWG “SBS” signature set^15^ (COSMIC_v3.3.1_SBS_GRCh38 from https://cancer.sanger.ac.uk/signatures/downloads/) if the cosine similarity between the two mutational profiles was at least 0.85. Further, all signatures were decomposed into the same set of PCAWG signatures using the python SigProfiler Assignment v0.0.24 tool (https://github.com/AlexandrovLab/SigProfilerAssignment), function decompose_fit.

In order to determine the extent to which the addition of Zou et al*.* and Kucab et al*.* samples influences the extracted SNV signature spectra and their respective exposures in our dataset, we implemented a subsampling approach whereby half of each external dataset is randomly removed thrice from the original dataset and the NMF algorithm is run on the remaining 117 samples (19 from Zou et al., 77 from Kucab et al*.* and 21 from our dataset), once for every subsampled set. Then, mutational signature spectra and exposures in our samples are compared to the original set.

For indel signatures, we used the SigProfiler Matrix Generator for R v1.2.4 tool to correctly classify the observed indels into one of the 83 categories defined in Alexandrov et al*.*^15^. Then, the SigProfiler ExtractorR v1.1.16 tool was launched on the same set of 214 samples from Kucab et al*.*, Zou et al*.*, and our experiments, using the generated mutation matrices. We considered the optimal solution given by the tool, resulting in a total set of 4 extracted indel signatures with high stability and low reconstruction error. As for the SNV signatures, we compared and decomposed our extracted spectra into the known PCAWG “ID” signature set^15^ (COSMIC_v3.3_ID_GRCh37.txt from https://cancer.sanger.ac.uk/signatures/downloads/).

### Regional enrichment analysis

We performed a regional enrichment analysis in order to estimate if the mutations detected in the irradiated clones were located in regions with a specific epigenetic pattern compared to the mutations detected in the non-treated clones. We tested three genomic features: the replication timing, the DNA repair histone mark H3K36me3 and the gene expression levels based on RNA-seq data. Replication timing bins, genomic bins extracted from chromatin mark H3K36me3, and regions based on variable gene expression were computed as in Supek and Lehner^28^. To detect significant association of mutations in specific genomic regions, we fitted a negative binomial regression using the glm.nb function from the MASS R package, on the counts of mutations per bins, controlling for the 3-nucleotide context and type of mutation in the case of SNV, as in Supek and Lehner^28^. The regional enrichment analysis source code is implemented using Nextflow^65^ and is available on the GitHub platform (https://github.com/tdelhomme/RegionalEnrichment-nf/).

### Statistical analyses

All the statistical analyses were performed using R software version 3.6.0. VariantAnnotation^66^ R package v1.32 was used to read the VCF files. The R packages BSgenome.Hsapiens.UCSC.hg38 v1.4.1 and BSgenome.Hsapiens.UCSC.hg19 v1.4.0 were used to load the human genome references inside R. MutationalPatterns^67^ R package v1.12 was used to generate the trinucleotide contexts. Ggraph v2.0.5 and Igraph v1.2.6 R packages were used to identify the cluster components. The statistical tests used to derive the p-values within some figures are described in the figure legends.

In order to systematically compare the mutation burdens and mutation signature exposures between all cell lines and treatments, we implemented a randomization test. Taking every mutation type (in the case of mutation burden comparison) and every signature (in the case of exposure comparisons), we randomly shuffled the clone labels (n = 21) 100,000 times. Then, we calculated the real mean mutation burden or exposure for each cell line (stratified by treatment and pooled) and for each treatment (stratified by cell line and pooled) and the mean from each shuffling iteration, resulting in 1 real mean and 100,000 randomised means for each comparison. Then, we calculated the pairwise difference between the mean of all cell lines and treatments (stratified or pooled) and compared the real mean difference to the distribution of randomised mean differences. For each such comparison, we calculated two empirical p-values^68^, where p = (r + 1)/(n + 1), r is the number of randomised differences > = or < = than the real mean difference and n is the total number of iterations (100,000). Then, all p-values from all comparisons across SNV signatures, ID signatures and mutation burdens were corrected (separately for each category) to account for the multiple comparisons problem using the p.adjust function from R core package stats v3.6.0, method “BH”/”fdr”^69^. Comparisons with an original p-value of 0.1 or lower were reported with their corrected values.

## Supplementary Information

Supplementary Figures.

Supplementary Figure 11.

Supplementary Figure 12.

Supplementary Figure 13.

Supplementary Table 1.

Supplementary Table 2.

## Data Availability

All Variant Call Format files (VCFs) derived from the WGS data generated in this study (see “Methods” for details) are available on FigShare repository using the following link: https://figshare.com/s/4f071118f2fde3f00a26.

## Code availability

Relevant source code for statistical analyses from this study is provided on GitHub at the following link: https://github.com/maia-munteanu/Radiation_paper_2023.
