# Supplementary figures and images for: Proton and alpha radiation-induced mutational profiles in human cells
Source: Sci Rep. 2023 Jun 16;13:9791. doi: 10.1038/s41598-023-36845-3 (PMC10275862; doi:10.1038/s41598-023-36845-3)

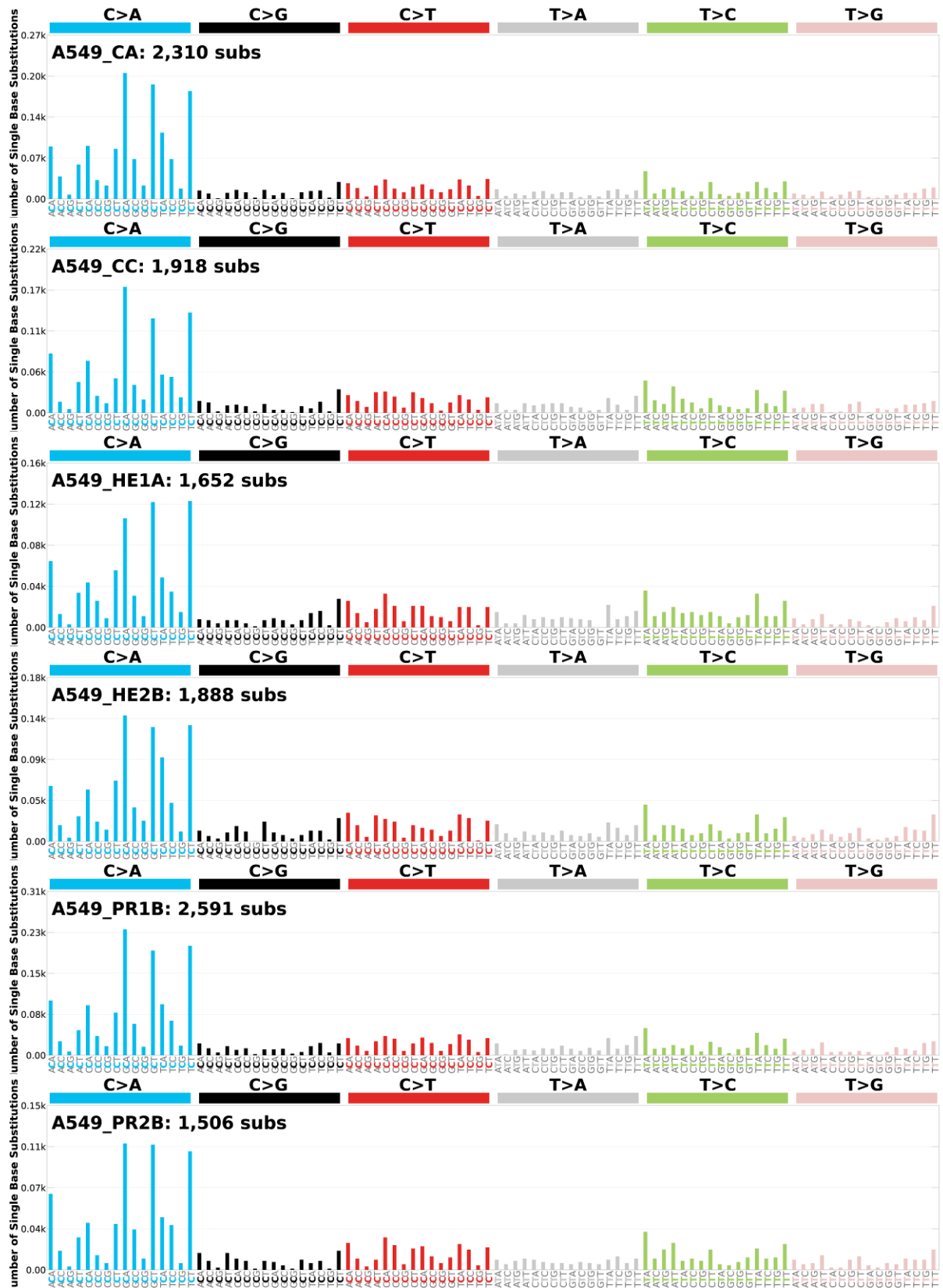

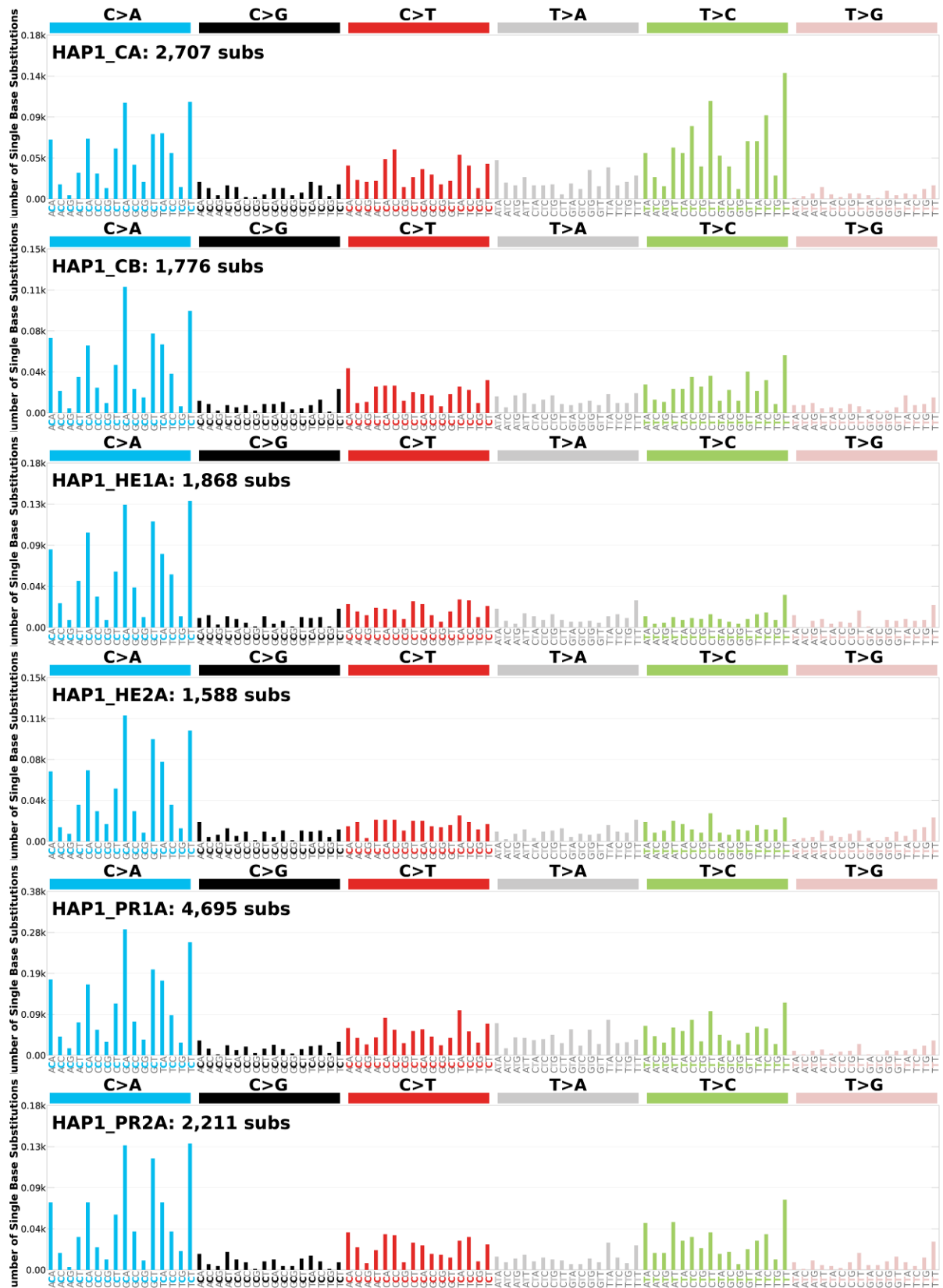

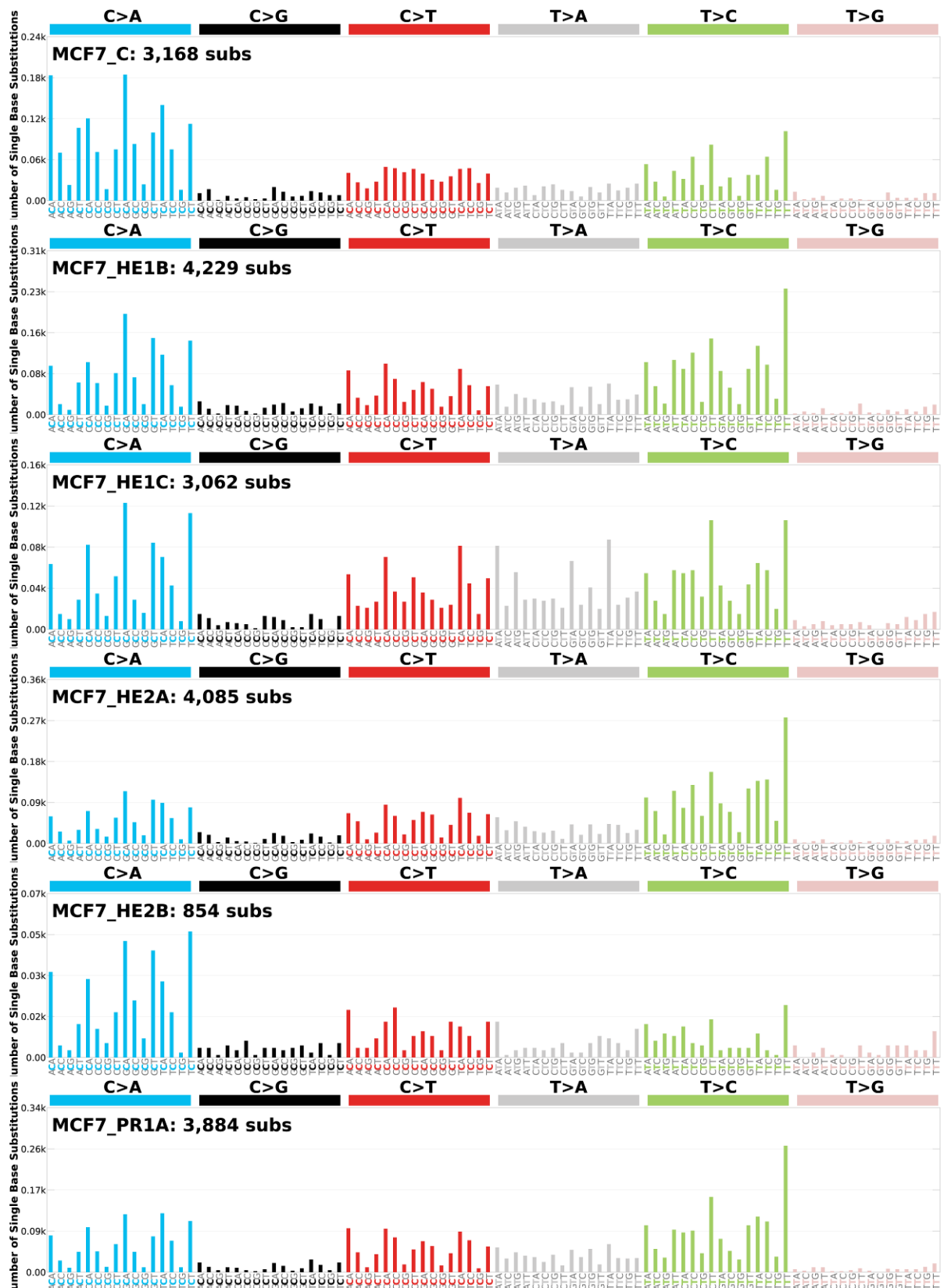

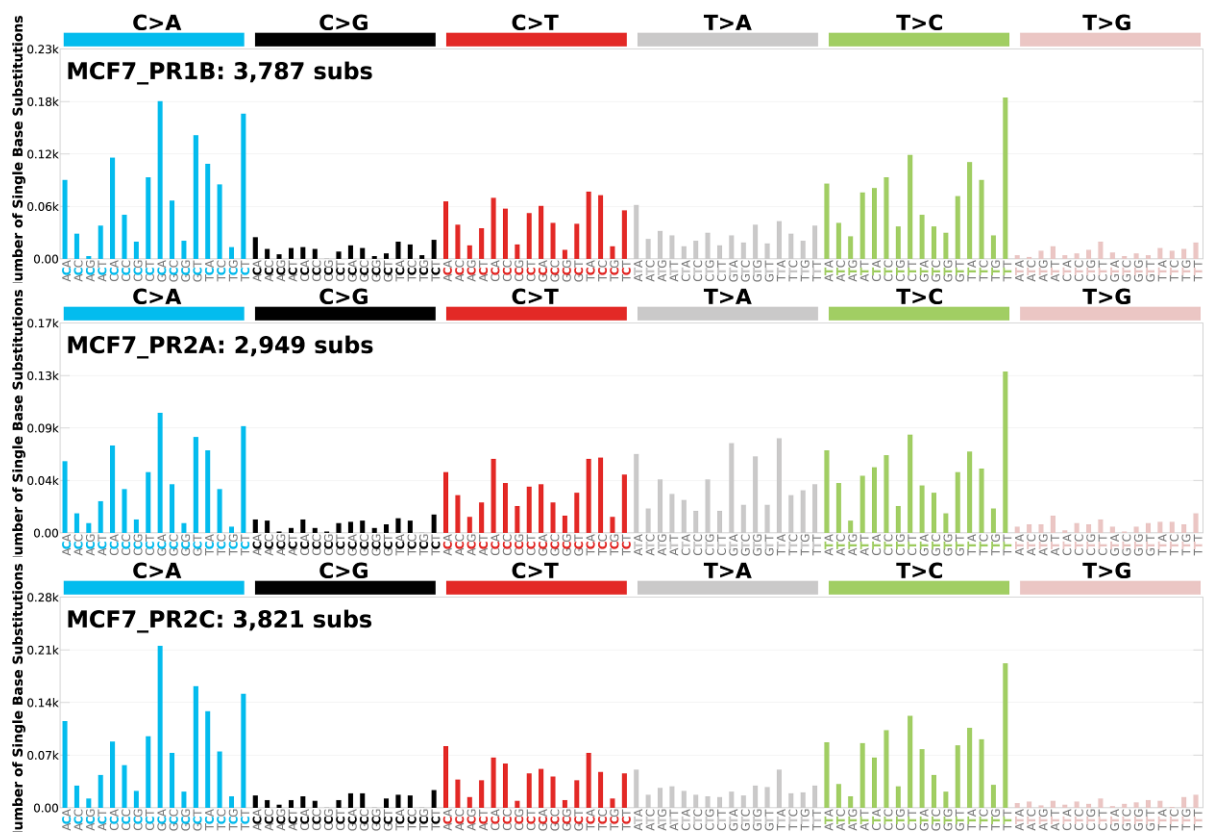

**Supplementary Figure 11.** 96-class spectrum of point mutations in each clone of our study.

Supplement: Supplementary file 2 — Supplementary Figure 11. [file 41598_2023_36845_MOESM2_ESM.pdf]

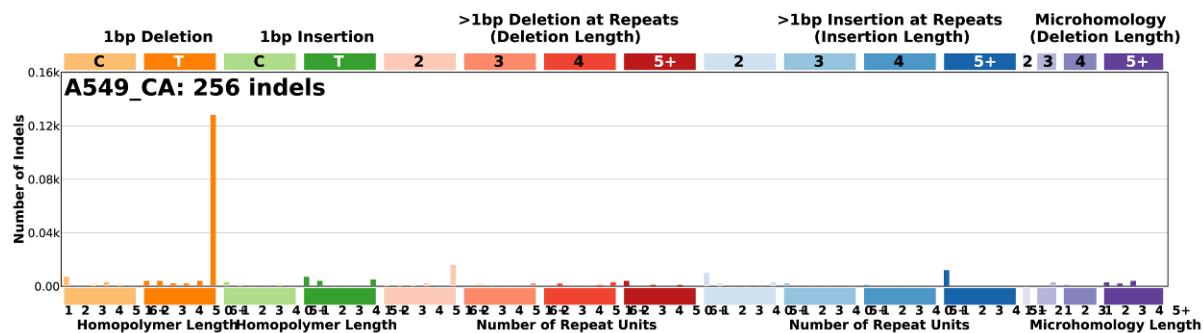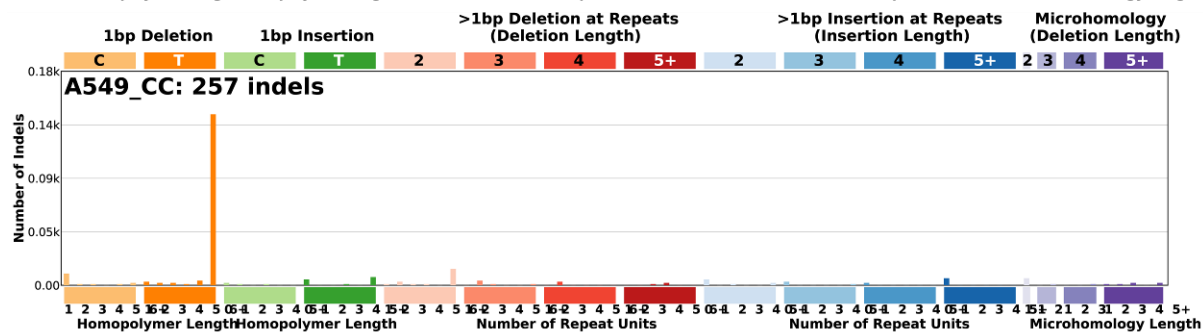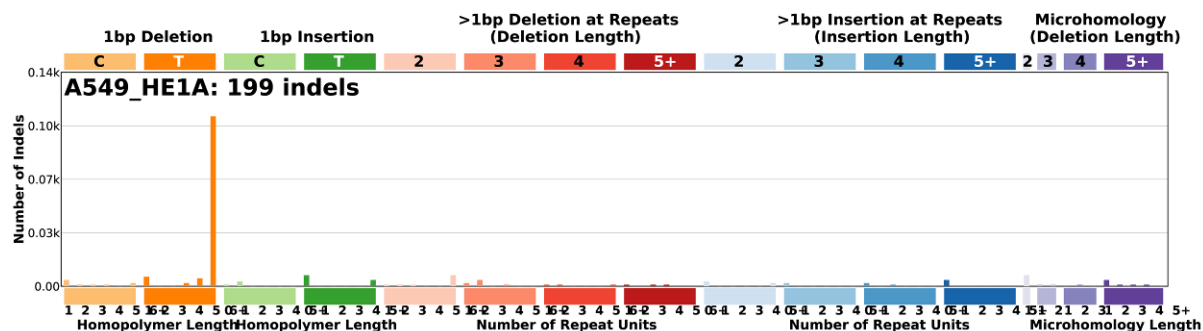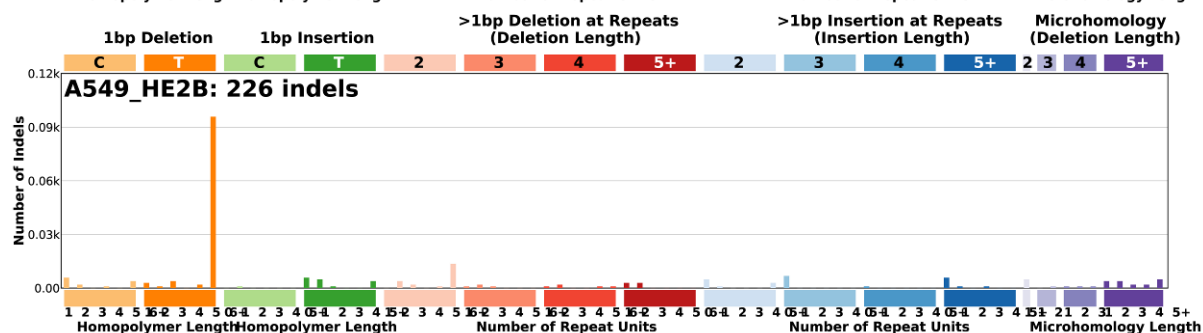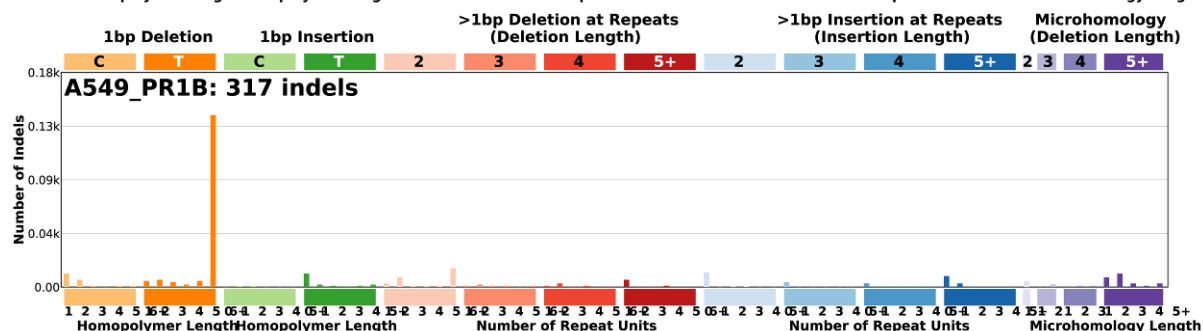

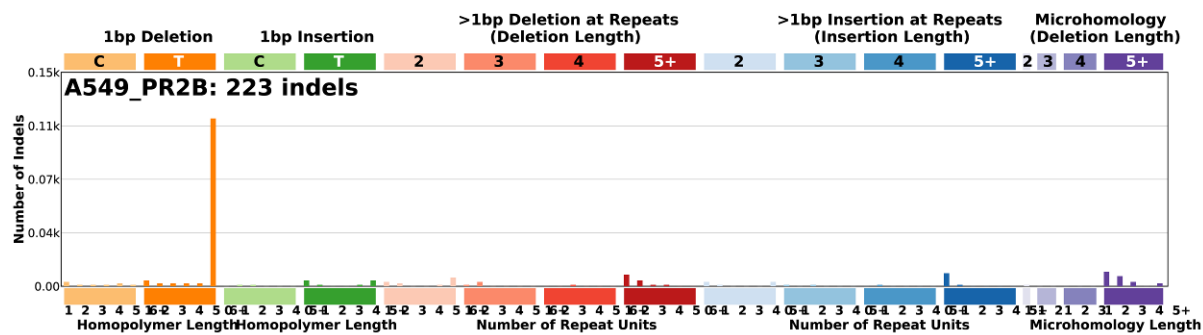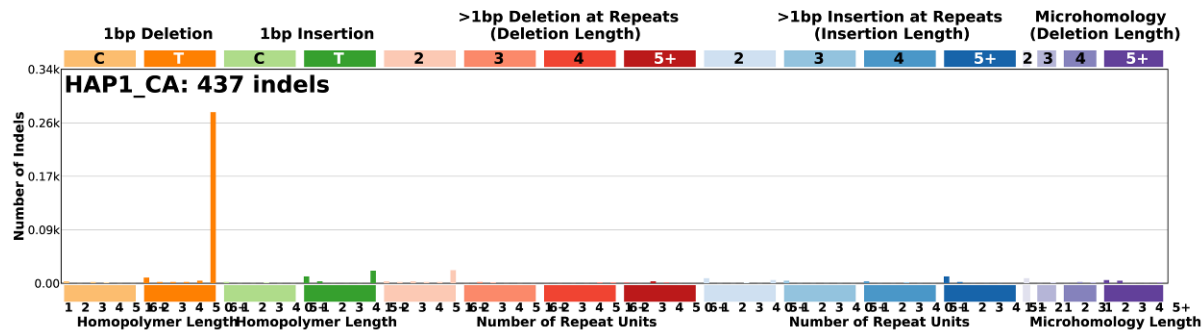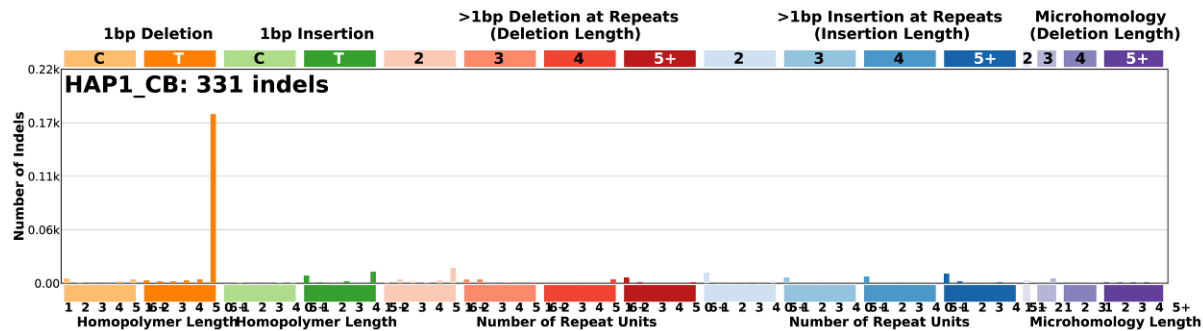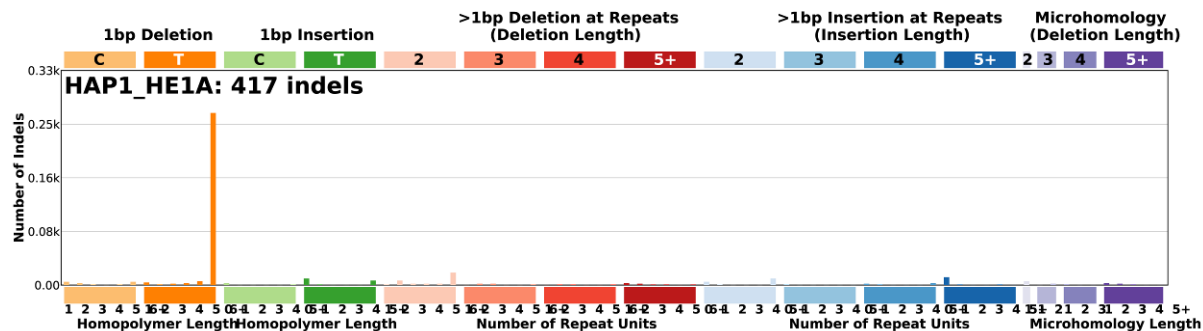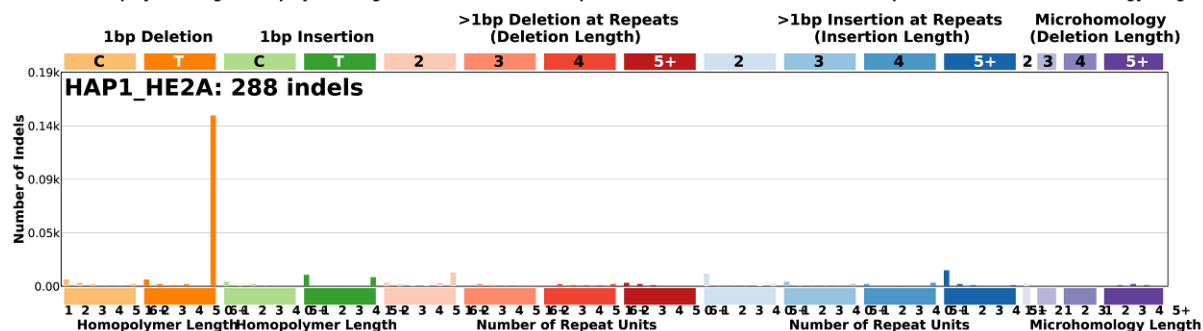

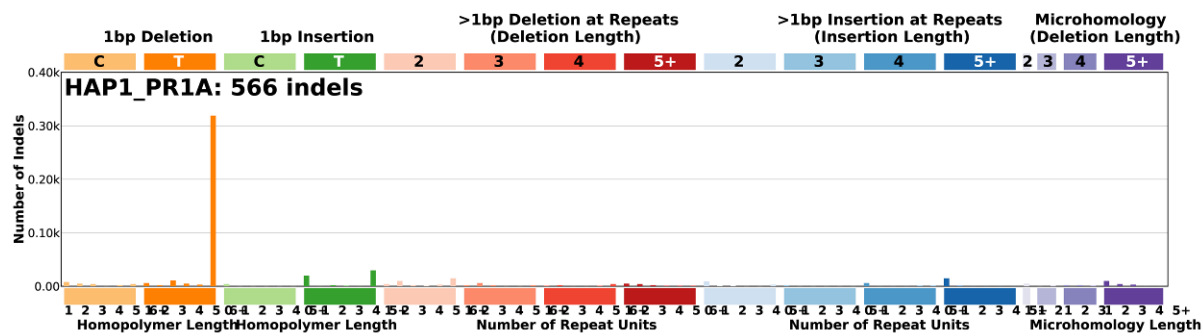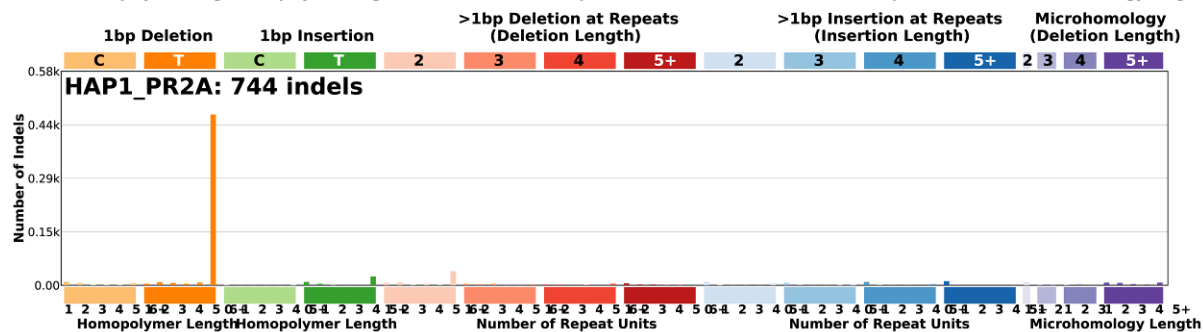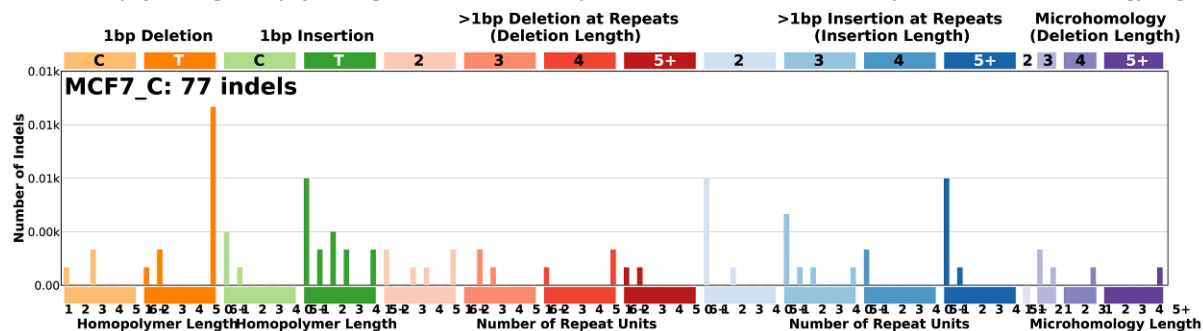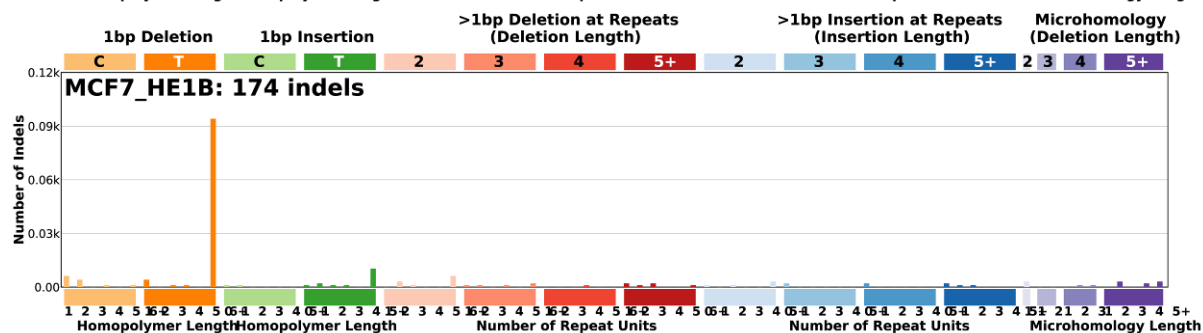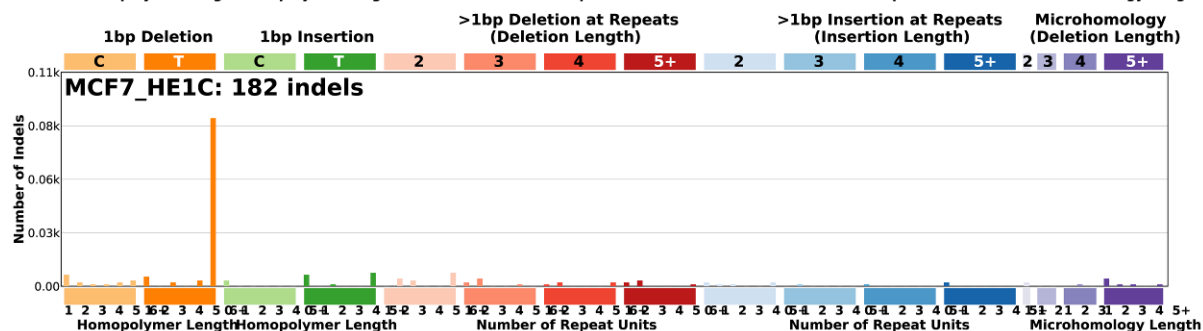

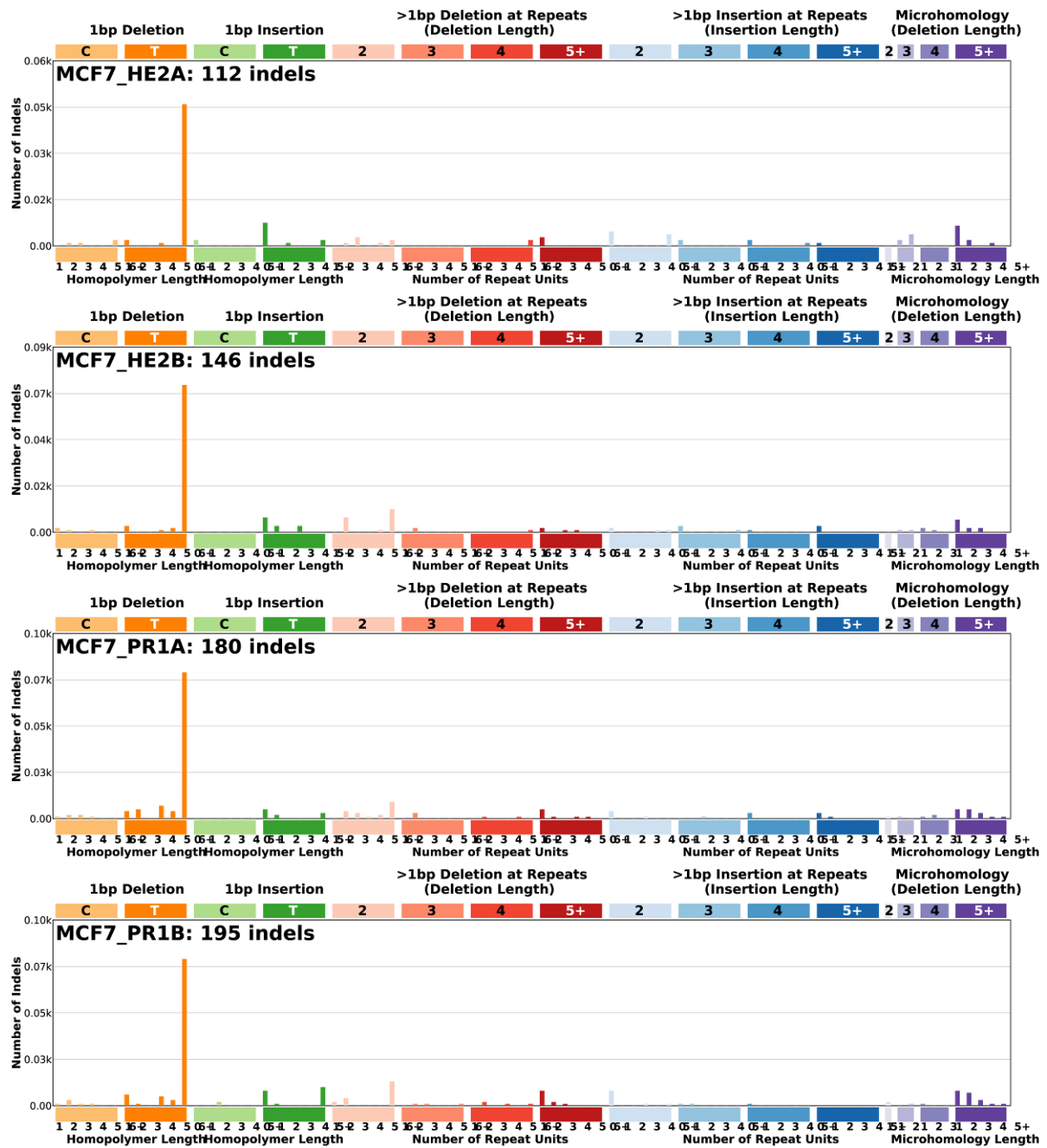

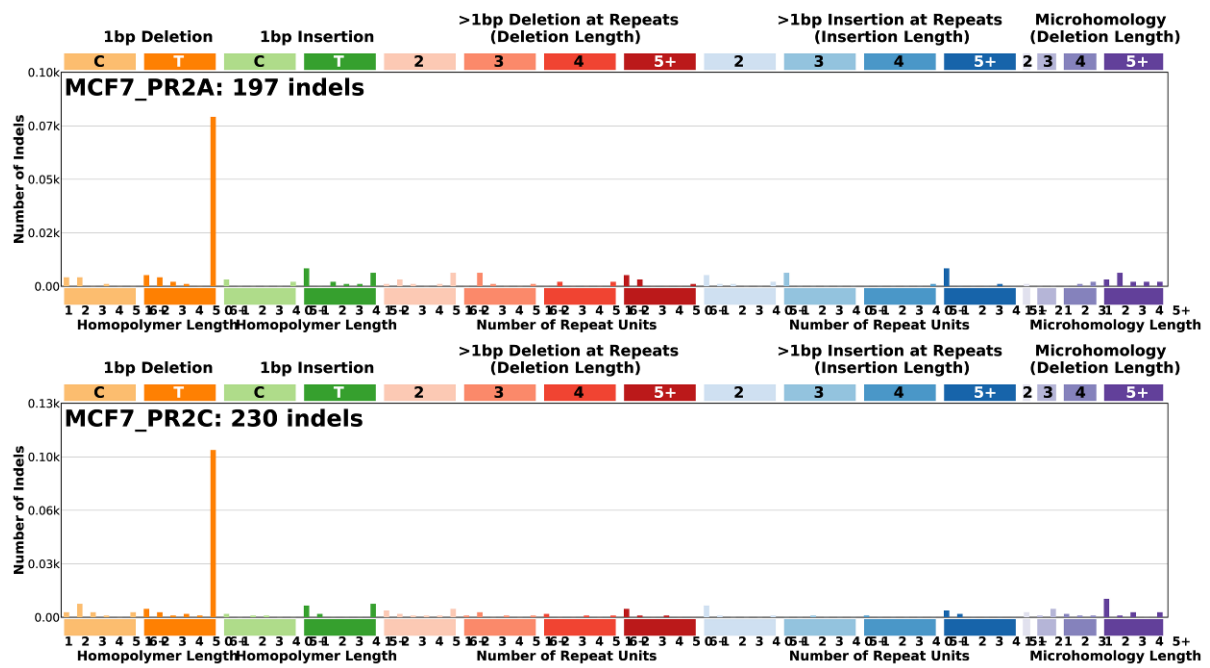

**Supplementary Figure 12.** 83-class spectrum of indel mutations in each clone of our study.

Supplement: Supplementary file 3 — Supplementary Figure 12. [file 41598_2023_36845_MOESM3_ESM.pdf]

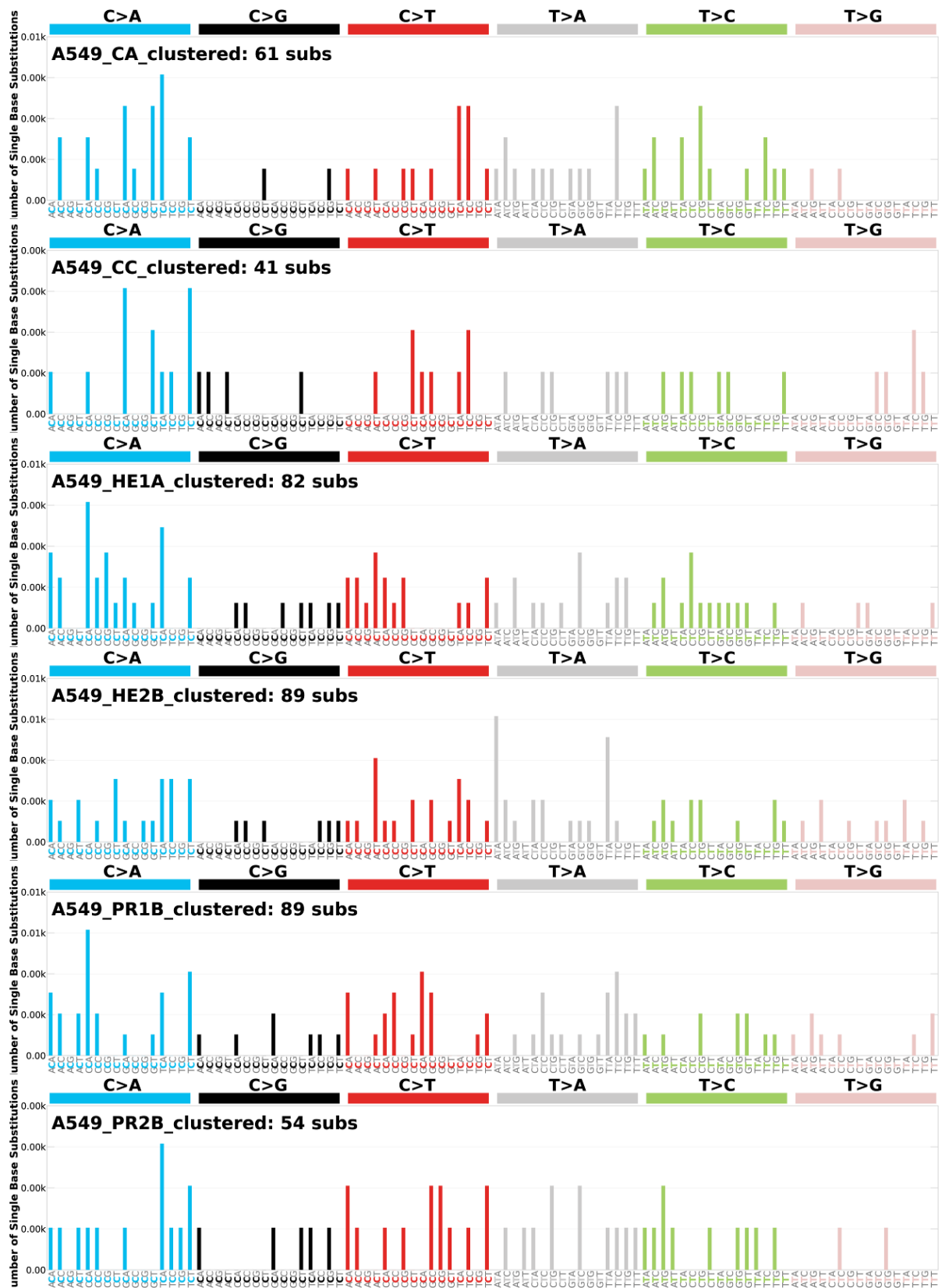

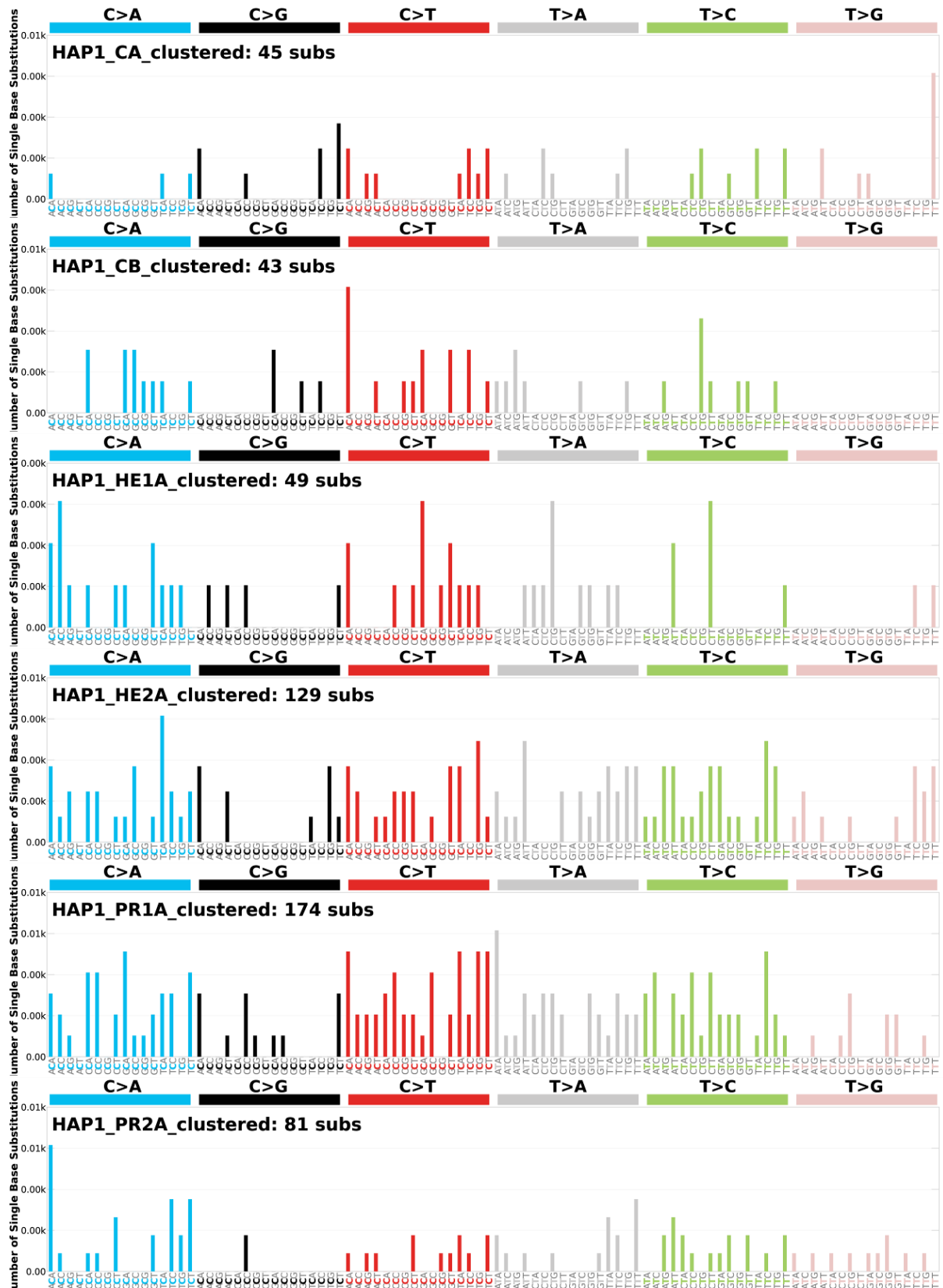

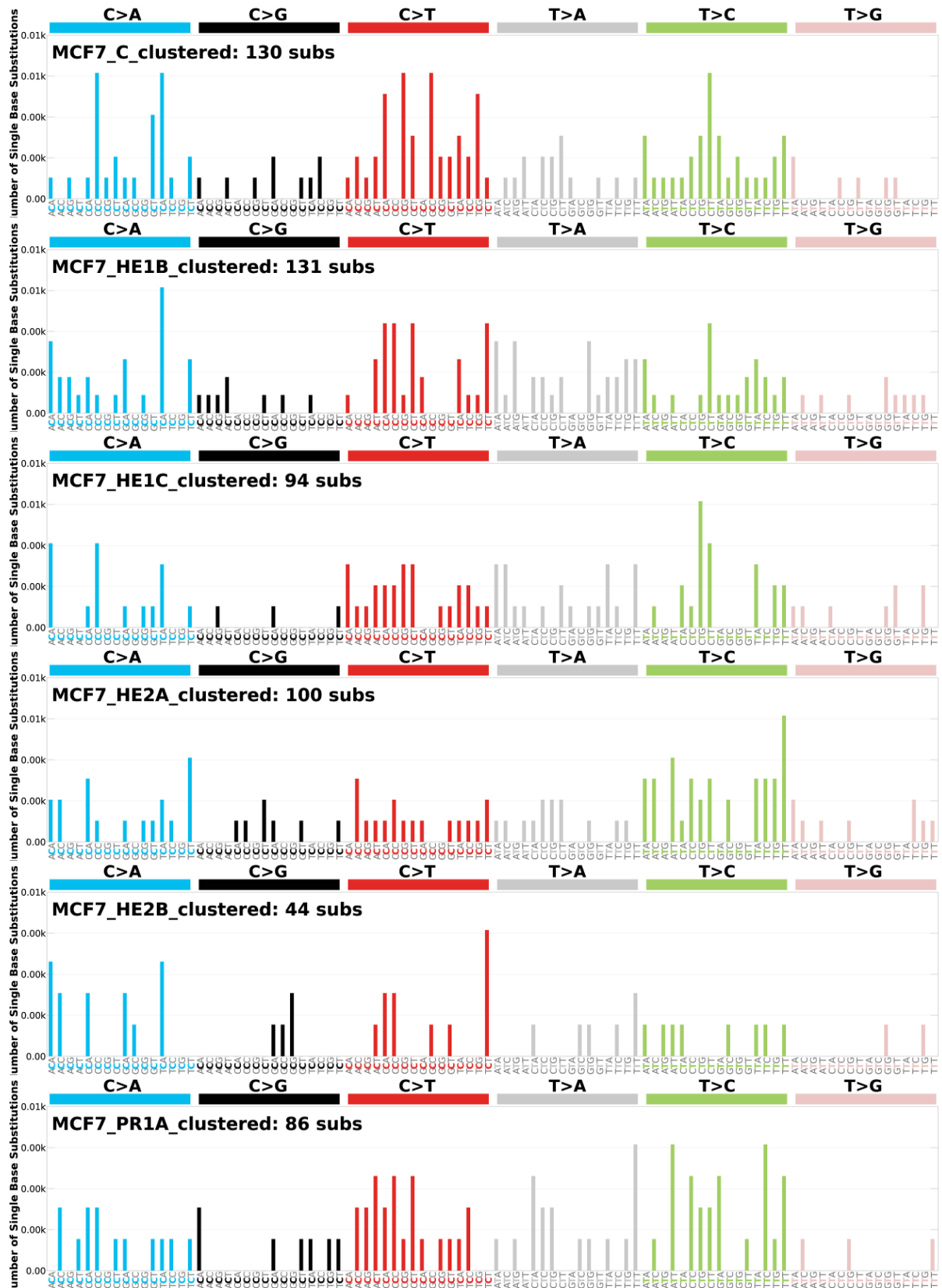

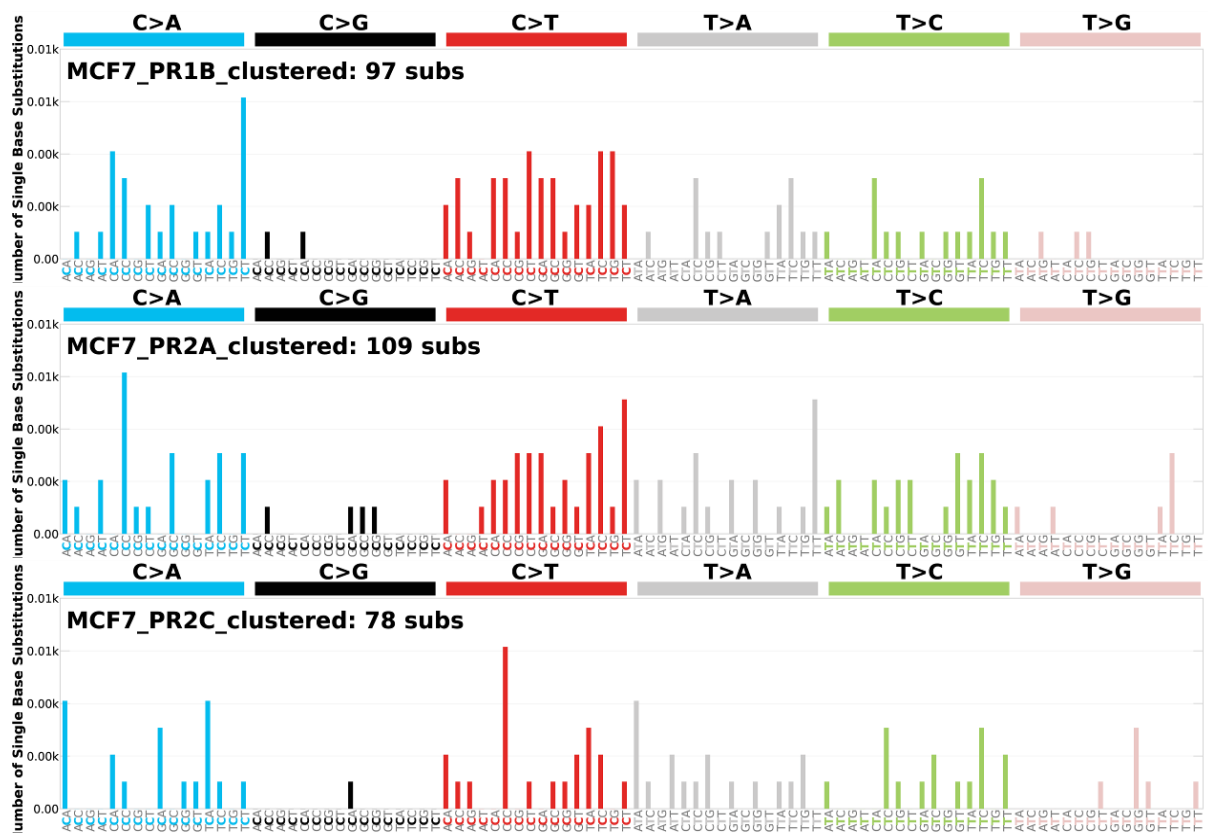

**Supplementary Figure 13.** 96-class spectrum of clustered point mutations in each clone of our study.

Supplement: Supplementary file 4 — Supplementary Figure 13. [file 41598_2023_36845_MOESM4_ESM.pdf]
